# SARS-CoV-2: Origin, Evolution, and Targeting Inhibition

**DOI:** 10.3389/fcimb.2021.676451

**Published:** 2021-06-17

**Authors:** Shuo Ning, Beiming Yu, Yanfeng Wang, Feng Wang

**Affiliations:** Key Laboratory of Molecular Medicine and Biotherapy, School of Life Science, Beijing Institute of Technology, Beijing, China

**Keywords:** SARS-CoV-2, evolution, pathogenic mechanism, structure, targeting inhibition

## Abstract

Severe acute respiratory syndrome coronavirus 2 (SARS-CoV-2) caused an outbreak in Wuhan city, China and quickly spread worldwide. Currently, there are no specific drugs or antibodies that claim to cure severe acute respiratory diseases. For SARS-CoV-2, the spike (S) protein recognizes and binds to the angiotensin converting enzyme 2 (ACE2) receptor, allowing viral RNA to enter the host cell. The main protease (Mpro) is involved in the proteolytic process for mature non-structural proteins, and RNA-dependent RNA polymerase (RdRp) is responsible for the viral genome replication and transcription processes. Owing to the pivotal physiological roles in viral invasion and replication, S protein, Mpro, RdRp are regarded as the main therapeutic targets for coronavirus disease 2019 (COVID-19). In this review, we carried out an evolutionary analysis of SARS-CoV-2 in comparison with other mammal-infecting coronaviruses that have sprung up in the past few decades and described the pathogenic mechanism of SARS-CoV-2. We displayed the structural details of S protein, Mpro, and RdRp, as well as their complex structures with different chemical inhibitors or antibodies. Structural comparisons showed that some neutralizing antibodies and small molecule inhibitors could inhibit S protein, Mpro, or RdRp. Moreover, we analyzed the structural differences between SARS-CoV-2 ancestral S protein and D614G mutant, which led to a second wave of infection during the recent pandemic. In this context, we outline the methods that might potentially help cure COVID-19 and provide a summary of effective chemical molecules and neutralizing antibodies.

## Introduction

SARS-CoV-2 is a highly contagious virus that has led to a worldwide epidemic; it causes fever, headache, cough, myalgia, fatigue, sputum production, and hemoptysis (Chen et al., 2020; Huang et al., 2020; Coronaviridae Study Group of the International Committee on Taxonomy of V, 2020; Wong, 2020; Wu and McGoogan, 2020; Xu J. et al., 2020). Most patients infected with SARS-CoV-2 can develop acute respiratory distress syndrome (ARDS) (Zumla et al., 2020). Until April 20, 2021, there have been 142,097,799 confirmed cases and 3,029,811 deaths globally. These statistics are rising rapidly, with approximately 10,000 deaths increasing per day. Some notable SARS-CoV-2 variants probably became dominant in many countries and caused uncontrolled transmission worldwide (Korber et al., 2020; Yurkovetskiy et al., 2020; Plante et al., 2021). According to the World Health Organization’s official report, SARS-CoV-2 has caused one of the worst global health emergencies in history. Until now, scientists are still struggling to prevent the spread of SARS-CoV-2 and have gained some improvements. In this review, we summarize the origin, evolution, and pathogenesis of SARS-CoV-2. In addition, we show the structures of spike protein, Mpro, and RdRp, as well as the complex structures of these three proteins with their inhibitors. In conclusion, we provide a summary of effective chemical molecules and neutralizing antibodies, which might potentially help cure COVID-19.

## Origin and Evolution of SARS-CoV-2

**Figure 1:**
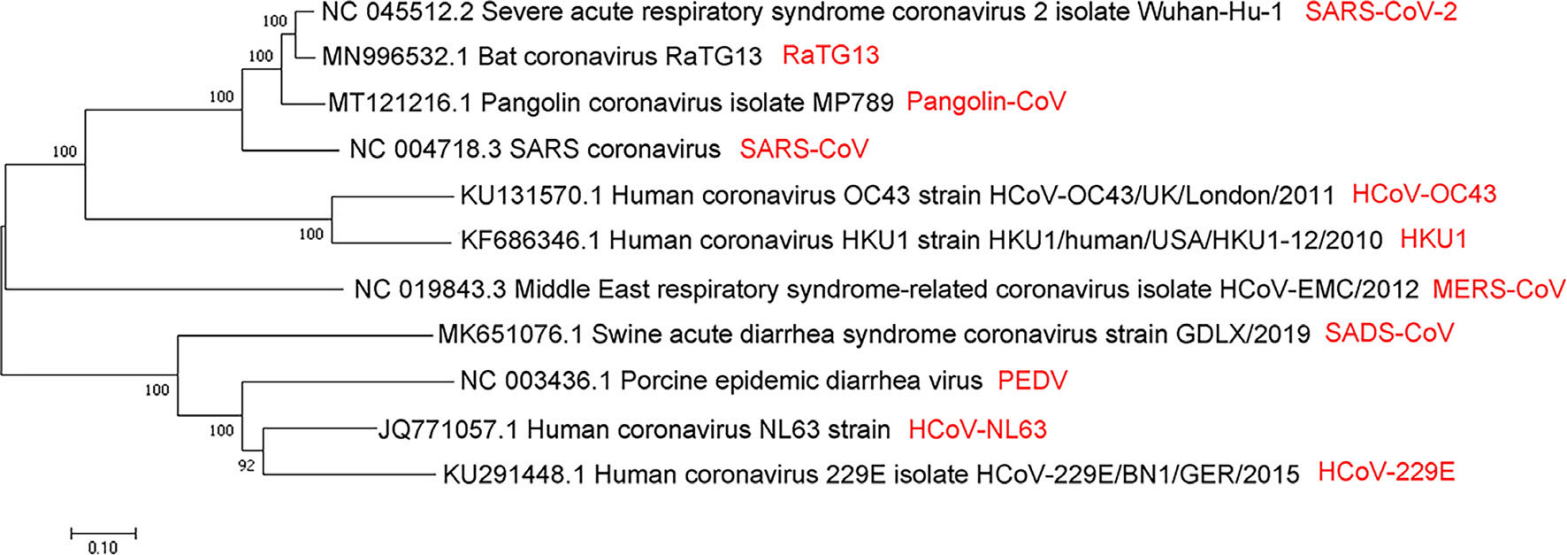
Evolutionary relationships of SARS-CoV, MERS-CoV, SARS-CoV-2, and several viruses from α- and β-coronavirus categories by the neighbor-joining method. A coronaviruses’ phylogenetic tree is shown based on the full-length genome sequence. All complete genome sequences of the coronavirus are downloaded from the NCBI reference sequence database RefSeq. The evolutionary relationship analysis involves 11 nucleotide sequences, including SARS-CoV, MERS-CoV, SARS-CoV-2, RaTG13, pangolin-CoV, HCoV-OC43, HKU1, SADS-CoV, PEDV, HCoV-NL63, and HCoV -229E. By using the neighbor-joining method, the evolutionary history was inferred. The optimal tree is shown, with the sum of branch length = 3.61257103. Next to the branches are shown the percentage of replicate trees (50 replicates) where the associated taxa are clustered together in the bootstrap test. The tree is drawn to scale, and its branch length is the same as the units used to infer the evolutionary distance of the phylogenetic tree. The phylogenetic tree is drawn by MEGA7.

Numerous studies claim that SARS-CoV-2 belongs to the group of coronaviruses, with a single-stranded genome of about 26-32kb (+ssRNA), which is the largest genome size of RNA virus as known (Woo et al., 2012). After a year of struggles, researchers have learned more about the origin, structure, and inhibition of SARS-CoV-2. Coronaviruses belong to the order Nidovirales, family Coronaviridae and the subfamily Coronavirinae. Coronavirinae consists of α-coronavirus, β-coronavirus, γ-coronavirus and δ-coronavirus (Woo et al., 2012). Coronavirus was first isolated from chickens in 1937. It was not until the SARS outbreak in February 2003 that the coronavirus was considered highly pathogenic to humans (Fouchier et al., 2003). Before that, the coronaviruses that spread among humans mainly caused mild infections in people with a weaker immune system (Vaheri et al., 2013; Su et al., 2016; Zhou et al., 2018). Since 2002, three zoonotic outbreaks have been caused by β-coronaviruses in China, SARS-CoV in 2003 (Fouchier et al., 2003), MERS-CoV in 2012 (Zaki et al., 2012), and the latest outbreak of SARS-CoV-2 at the end of 2019 (Cauchemez et al., 2013; Cui et al., 2019; Huang et al., 2020). Apart from these, there are other four human coronaviruses: HCoV-229E, HCoV-OC43, HCoV-NL63, and HKU1. These coronaviruses could lead to mild diseases in the upper respiratory of the immune-competent hosts. Regardless, some of viruses could induce serious infections in infants, children, and the elderly (Su et al., 2016). Some people suspect that SARS-CoV-2 directly originated from SARS-CoV, while others believe that it leaked from the laboratory (Andersen et al., 2020). However, the evolutionary analysis of SARS-CoV-2, SARS-CoV, and MERS-CoV by neighbor-joining method showed that SARS-CoV-2 belongs to a new evolutionary branch of coronaviruses (**Figure 1**) (Cao et al., 2005; Chan et al., 2015; Jiang et al., 2020; Rabaan et al., 2020). Comparing the sequences of SARS-CoV and SARS-CoV-2, the homology is about 90%. Notably, the SARS-CoV-2 spike (S) protein has high sequence homology (up to 98%) with that of bat coronavirus RaTG13 (Li et al., 2003). However, the amino acid sequence homology between MERS-CoV and SARS-CoV-2 related virus is less than 90% (Chan et al., 2015). Apart from the amino acid sequence, the receptor and the host of MERS-CoV are distinct from SARS-CoV-2 and SARS-CoV (Hamming et al., 2007; Chen et al., 2013; Xia et al., 2014; Jiang et al., 2014; Forni et al., 2017; Chafekar and Fielding, 2018; Song et al., 2019; Bleibtreu et al., 2020) (**Figure 1**).

**Figure 2:**
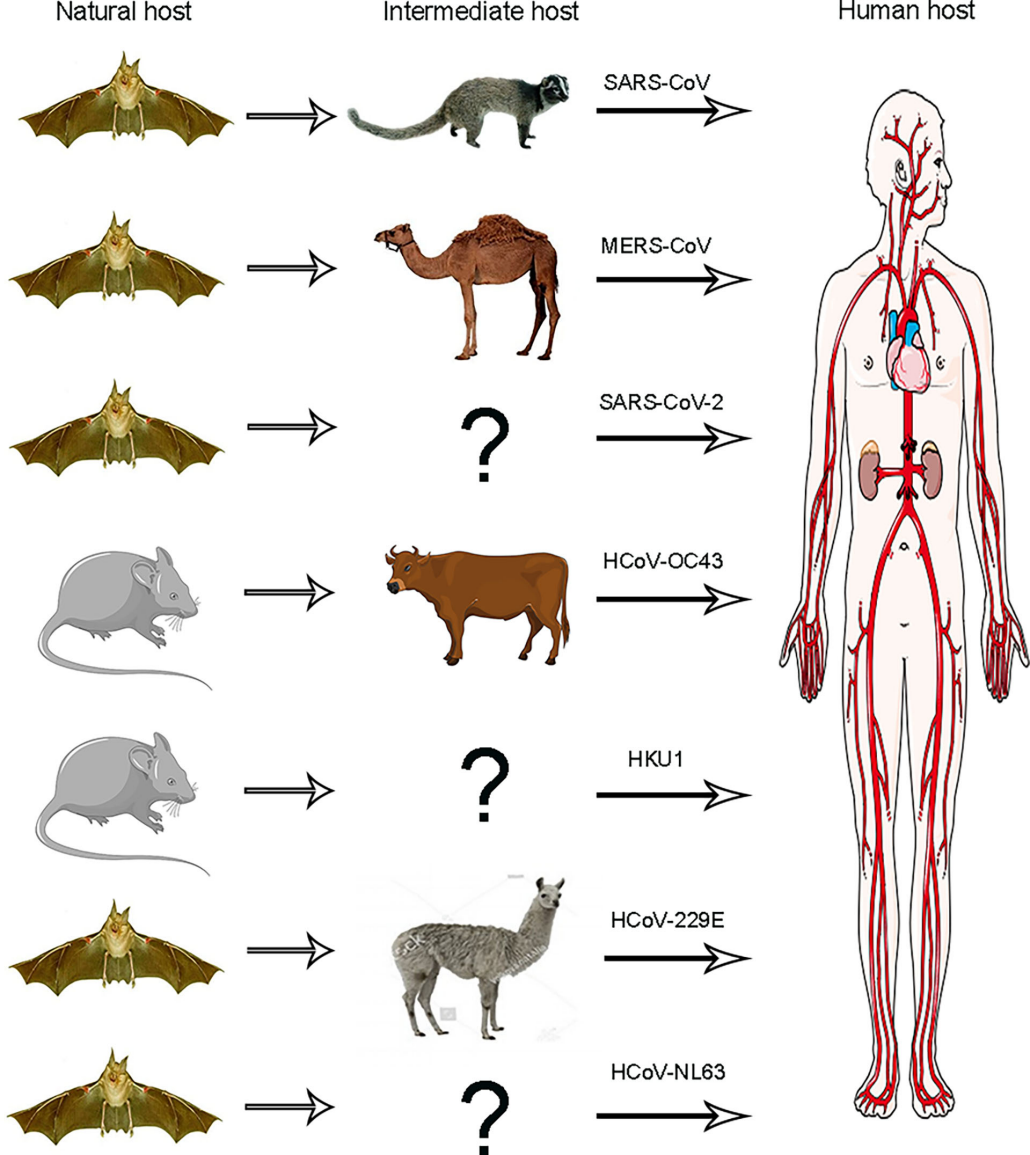
Animal origins of human coronaviruses. SARS-CoV, MERS-CoV, and SARS-CoV-2 originated from bats; HCoV-229E and HCoV-NL63 usually lead to mild infections in immune people. The origins of these viruses have recently been found in African bats, and camelids may be intermediate hosts of HCoV-229E, HCoV-OC43, and HKU1, all of which are also harmless in humans and probably originated from rodents.

Some researchers believe that some viruses originated from rodents, such as HCoV-OC43 and HKU1; others originated from bats, which consist of HCoV-NL63, HCoV-229E SARS-CoV, and MERS-CoV (Mittal et al., 2020) (**Figure 2**). However, the origin of SARS-CoV-2 remains unknown. Some people suspect that bats might be the SARS-CoV-2’s source because the RaTG13 virus isolated from bats has high sequence similarity with SARS-CoV-2 that has come from the same branch. A study reported that bat RaTG13 and SARS-CoV-2 recognize the same receptor ACE2 and have the same ability to infect cells through this receptor. These studies also identified that the ACE2 binding ridge in the bat RaTG13 receptor binding motif (RBM) contains four residues, which is the same as SARS-CoV-2. Moreover, Leu455, conserved on SARS-CoV-2 and RaTG13, may contribute to the recognition of ACE2. Thus, these findings suggest that SARS-CoV-2 may be derived from RaTG13 or shares a common ancestor with RaTG13 (Li et al., 2003; Shang et al., 2020).

## The Pathogenic Mechanism of SARS-CoV-2

**Figure 3:**
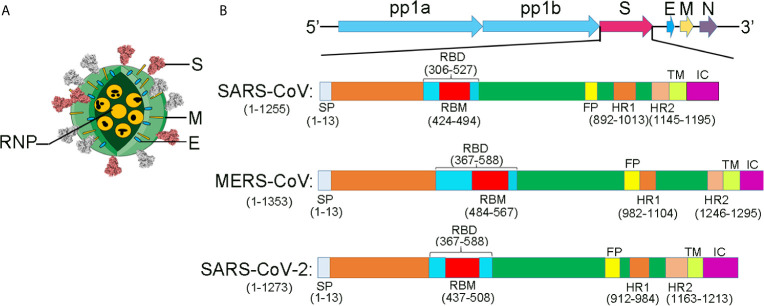
The comparison of the structural proteins of SARS-CoV, MERS-CoV, and SARS-CoV-2. **(A)** Coronavirus virion structure. Coronaviruses, forming enveloped and spherical particles, are with a diameter of 100–160 nm. The positive-sense, single-stranded RNA (ssRNA) genome is 27–32 kb in size. RNP, ribonucleoproteins; S, spike; E, small membrane envelope; M, membrane are transmembrane proteins. **(B)** The schematic diagram for the components of the SARS-CoV, MERS-CoV, and SARS-CoV-2. A polyprotein, pp1ab, which is encoded at two-thirds of the 5’-terminal of the genome, is further cleaved into 16 non-structural proteins. These proteins are involved in transcription and replication. The structural proteins, envelope glycoproteins spike (S), envelope (E), membrane (M), and nucleocapsid (N), are encoded at the 3’ terminus.

The diameter of the SARS-CoV-2 virus particle is approximately 100 nm (**Figure 3**). It contains structural proteins (the membrane (M), envelope (E), spike (S), and nucleocapsid (N) proteins), an accessory protein, and non-structural proteins (nsps) (NSP1–16) (Cao et al., 2005; Chang et al., 2014; Lu et al., 2020; Yao et al., 2020b; Wu et al., 2020a). The SARS-CoV-2 resembles a corona because of the plenty of S proteins on the envelope. The S protein of coronaviruses, which could help to binding, fuse membrane, enter into the host cells, and induct antibody, is a type I transmembrane (TM) glycoprotein. The accessory and non-structural proteins’ functions are related to virulence because those proteins are involved in viral replication and assembly (Holmes, 2003).

**Figure 4:**
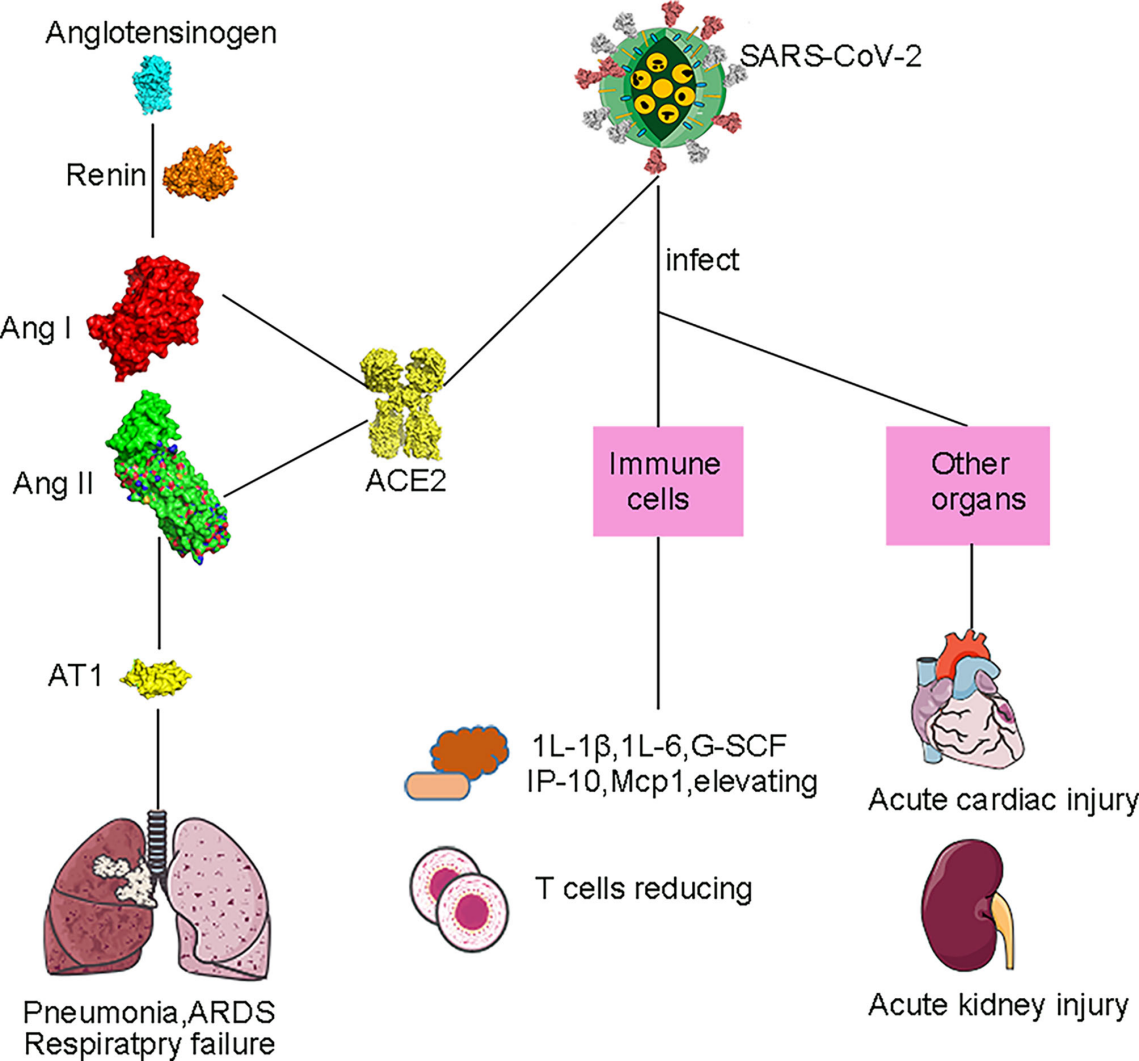
Pathogenesis of SARS-CoV-2. By inhibiting the ACE2’s function, SARS-CoV-2 could cause the injury of the lung. ACE2 plays an important role in the renin-angiotensin system (RAS). Firstly, renin converts angiotensinogen into angiotensin I (AngI), and then AngI is converted into AngII under the ACE2’s action. ACE2 down-regulates the AngI’s and Ang II’s levels. Then, the Ang II type I (AT1) receptors are bound by Ang II, causing lung injury, which includes acute lung injury, pulmonary fibrosis, and pulmonary hypertension. At the same time, Ang II can directly induce rapid growth or proliferation of pulmonary artery smooth muscle cells through AT1, thereby causing pulmonary hypertension. Besides the intestines and lungs, the heart, kidney, esophagus, bladder, ileum, testis, and adipose tissue also could express ACE2, and its level is higher than that of the lung. In addition, tumor tissues have high ACE2 expression, making cancer patients more vulnerable than others.

SARS-CoV-2 invades cells in two ways. One is interfering with target cells directly. SARS-CoV-2 occupies ACE2 on the membrane (Kuba et al., 2006; Wu et al., 2011; Hemnes et al., 2018; Gao et al., 2019), blocking signals, disturbing the renin-angiotensin (RAS) system. Another is subsequent immune system dysfunction (Guo et al., 2008). After SARS-CoV-2 occupies ACE2, a decrease in the number of immune cells is observed, and IL-1 levels are elevated (Chen et al., 2020) (**Figure 4**).

SARS-CoV-2 employs S protein to infect target cells by fusing the membranes (Tian et al., 2020; Walls et al., 2020; Yan et al., 2020). S protein consists of S1 and S2 subunits. An N-terminal domain (NTD), a receptor-binding domain (RBD), and a C-domain are the main domains of the S1 subunit. Moreover, RBM of the RBD, located in a C-domain’s accessory subdomain, is a hypervariable region for receptor recognition. Another subunit, including two 7-valent element repeats (HR1 and HR2) and a fusion peptide (FP), could fuse to the cell membrane (Bosch et al., 2003; Belouzard et al., 2009; Burkard et al., 2014; Millet and Whittaker, 2014; Millet and Whittaker, 2015; Kirchdoerfer et al., 2016; Park et al., 2016; Walls et al., 2016). In this process, SARS-CoV-2 is more infectious than SARS-CoV because the free energy of binding between RBD and ACE2 of SARS-CoV-2 is remarkably lower than that of SARS-CoV (Wang K. et al., 2020).

**Figure 5:**
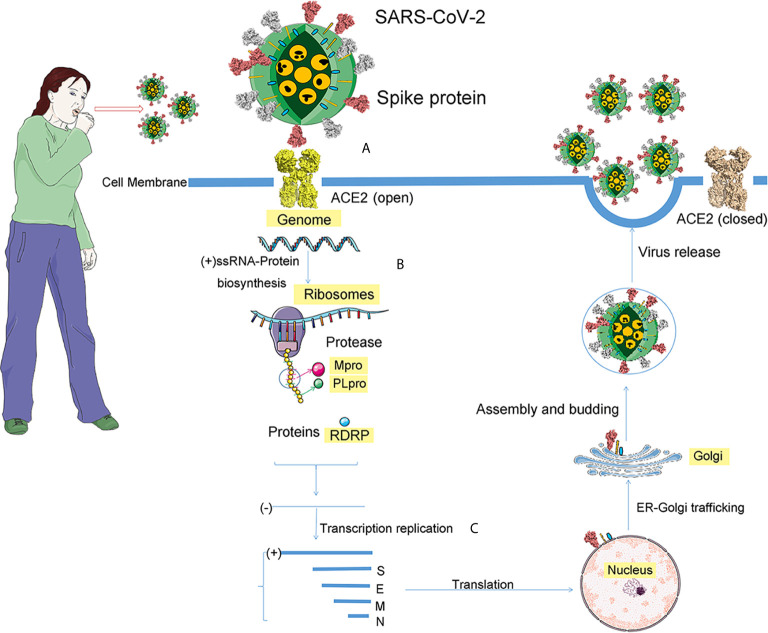
The process of SARS-CoV-2 infection. After coughing, the infected patient will spread virus particles into the air, and people will be exposed to these virus particles. If these virions reach the cell, the infection process begins. The infection is divided into three steps. **(A)** For SARS-CoV-2, the receptor of spike protein is ACE2. The pink ball is the spike protein. **(B)** After entering the cell, the positive genomic RNA attaches to the host ribosome, translating two large coterminal polyproteins. These proteins are treated by proteolysis into components that package new virions. The two proteases involved in this proteolysis process are the major coronavirus protease (Mpro) and the papain-like protease (PLpro). **(C)** RNA-dependent RNA polymerase (RdRp), a replicase encoded by SARS-CoV-2, replicates the RNA genome.

After the SARS-CoV-2 entering the host cell, the viral RNA is attached to the ribosome of host, translating two large, coterminal polyproteins. Subsequently, these proteins were digested into components by proteolysis for packaging new virions. Two proteases for this proteolysis are the papain-like protease (PLpro) and the coronavirus main protease (Mpro). Like other known coronaviruses, SARS-CoV-2 also employs RNA-dependent RNA polymerase (RdRp) to replicate the genome of RNA. Above all, the four proteins, spike, Mpro, PLpro, and RdRp, are essential to virus assemble and pathogenesis (**Figure 5**) (Sheahan et al., 2020).

Given the crucial role of S protein and ACE2 in receptor recognition, as well as the critical role of Mpro and RdRp in the SARS-CoV-2’s replication and transcription, interfering with their functions may provide effective antiviral strategies (Hoffmann et al., 2020). In other words, therapeutics currently targeting structural protein (S protein), non-structural proteins (Mpro, and RdRp) from SARS-CoV-2, and ACE2 from the host are potential treatment options for SARS-CoV-2. Recently, studies on the structural biology of the SARS-CoV-2 and structure-based antibody and drug discovery have appeared. We review these findings in the following sections.

## Structural Protein andIts Inhibitors

### Structure of SARS-CoV-2 Spike Protein

**Figure 6:**
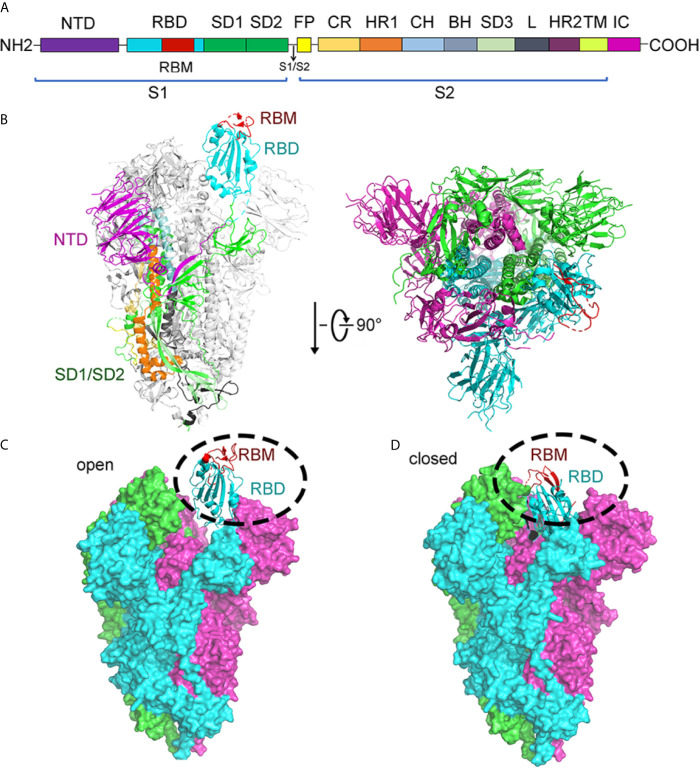
Structure of SARS-CoV-2 spike protein. **(A)** Schematic of the SARS-CoV-2 spike protein primary structure colored by domain. NTD, N-terminal domain; RBD, receptor binding domain; RBM, receptor binding motif; SD1, subdomain 1; SD2, subdomain 2; FP, fusion peptide; CR, connecting region; HR1, heptad repeat 1; CH, central helix; BH, β-hairpin; SD3, subdomain 3; HR2, heptad repeat 2; TM, transmembrane domain; IC, cytoplasmic tail. Arrows denote protease cleavage sites. **(B)** From the side view and top view, the Cryo-EM structure of the SARS-CoV-2 spike in the prefusion conformation shows that a single RBD is in the up conformation and other RBDs are in the down conformation in either white or gray. The RBD, which in up conformation, is shown in ribbons colored corresponding to the schematic in **(A)**. **(C)** Cryo-EM structure of the SARS-CoV-2 spike in the open state. There is one open RBD domain. The three chains are depicted in green, cyan, and magnet, respectively. RBD and RBM are shown in cyan and red cartoon, respectively. **(D)** Cryo-EM structure of the SARS-CoV-2 Spike in the closed state. The three chains are depicted in green, cyan, and magnet, respectively. RBM is colored in red. RBD and RBM are shown in cyan and red cartoon, respectively. All structures are drawn by Pymol.

The structure of S protein was resolved in two states: open and closed state (Walls et al., 2020; Wrapp et al., 2020). In the open state of S protein, one RBD was in the “up” conformation and other RBDs in the “down” conformation. The RBD domain (in “up” conformation) exhibits hinge-like conformational movement, which may be the essential determinant for binding host receptor ACE2 (**Figures 6A–C**). Meanwhile, to fuse the membranes of the virus and host cell, the S protein undergoes a drastic structural rearrangement. In the closed state of S protein, all three RBDs in the “down” conformation are at the interface among protomers (Walls et al., 2020). Compared to the “up” conformation, the “down” conformation in the close state is near the trimer’s central cavity (**Figure 6D**). Thus, it is expected that the opening of the SARS-CoV-2 RBD is essential for interacting with ACE2 and triggering changes of conformation. The opening of RBD results in cleaving the S2 site, fusing membrane and entering into cells.

After the SARS-CoV-2 pandemic, 12,379 single nucleotide polymorphisms (SNPs) are downloaded in genomic data on June 25, 2020. In these SNPs, four SNPs, C3037U, C14408U, A23403G, and C241U, show high frequency. As for the A23403G, it encodes the mutant D614G in Spike protein. In late January 2020, D614G was first discovered in the viral genomes. Compared with the virus strain provided by Wuhan, Spike mutant D614G is caused by the 23,403nucleotide mutation of SARS-CoV-2. The G614 form is rare in the world, but it has attracted much attention. However, the transition of spike mutants from D614 to G614 occurred all over the world (Korber et al., 2020). Mutations that increase the chance of viral infection occurred mainly in S protein (Plante et al., 2021). With the sequencing analysis, studies showed that the S mutant D614G is a pseudo-typed virion. Because mutant D614G has association with a lower RT-PCR cycle threshold among patients, the upper respiratory tract has high viral load (Korber et al., 2020). Animal experiments also confirm these results. Hamsters infected with D614G have higher viral titers in the trachea and nasal wash than those in the lungs. This also supports the mutant D614G increase the load of virus in the Covid-19 patients’ upper respiratory tract and might enhance the spread (Plante et al., 2021). Besides, the ratio of the open conformations of mutant D614G increased up to 58% and is in sharp contrast in the ancestral SARS-CoV-2 virus, in which the ratio of RBDs in the open conformation is only 18%. In comparison with the ancestral S protein in the closed state, the NTD and the INT domain of the S protein D614G shift by 4 Å and 6 Å, respectively. Moreover, the NTD of the S protein D614G has a 3Å shift in the open state (**Figure S1**) (Yurkovetskiy et al., 2020).

**Figure 7:**
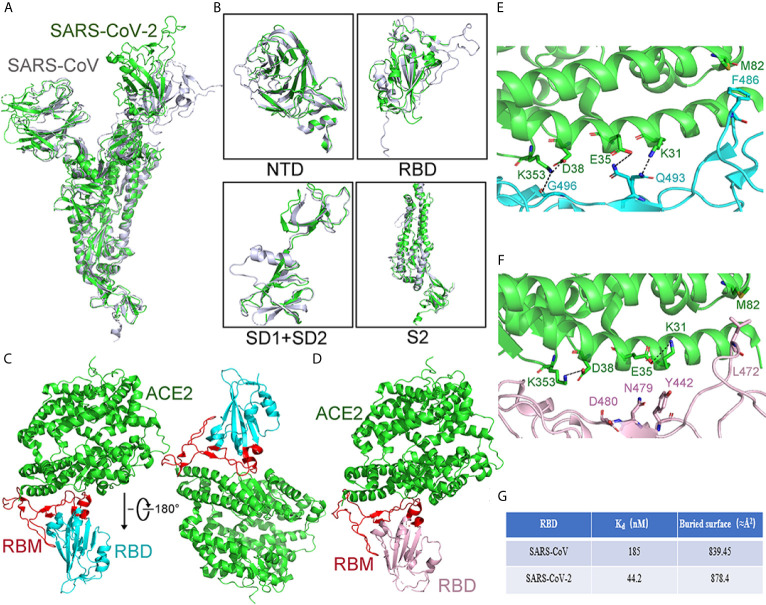
Comparison of the S protein in SARS-CoV-2 and SARS-CoV. **(A)** Compared whole S protein between SARS-CoV-2 and SARS-CoV. SARS-CoV-2 and SARS-CoV are shown in green and gray, respectively. **(B)** Compared different domains of S protein between SARS-CoV and SARS-CoV-2. Structural domains from SARS-CoV-2 S protein have been aligned with the domain from SARS-CoV S as follows: NTD (top left), RBD (top right), SD1, and SD2 (bottom left), and S2 (bottom right). SARS-CoV and SARS-CoV-2 are shown in gray and green, respectively. **(C)** The overall structure of the SARS-CoV-2 RBD with ACE2 from the side view. ACE2 is shown in green. The SARS-CoV-2 RBD is shown in cyan and RBM in red. **(D)** The overall structure of the SARS-CoV RBD with ACE2 from the side view. The SARS-CoV RBD is shown in pink and RBM in red. ACE2 is shown in green. **(E)** The structural details of the interface between ACE2 and SARS-CoV-2 RBM. The interface between the SARS-CoV-2 RBM and ACE2 has been shown. The SARS-CoV-2 RBM is shown in cyan. ACE2 is shown in green. **(F)** The structural details of the interface between ACE2 and SARS-CoV RBM. The interface between SARS-CoV RBM and ACE2 has been shown. ACE2 is shown in green. The SARS-CoV RBM is shown in pink. **(G)** Compared Kd value and buried surface between the SARS-CoV RBD or SARS-CoV-2 RBD with ACE2. The value buried area is calculated as the average value. All structures are drawn by Pymol.

The structures of the S proteins between SARS-CoV-2 and SARS-CoV are similar, and the root mean square deviation (RMSD) on 959 Cα atoms is 3.8 Å. The overall structure of SARS-CoV-2 S protein resembles that of SARS-CoV S protein, with a root mean square deviation (RMSD) of 3.8 Å over 959 Cα atoms (**Figure 7A**) (Wrapp et al., 2020). For the RMSD value, the structural comparison of S2 subunits, NTDs, subdomains 1 and 2 (SD1 and SD2), and RBDs are 2.0Å, 2.6Å, 2.7Å, and 3.0Å, respectively (**Figure 7B**). The largest discrepancy exists in the RBDs “down” conformations. The “down” RBD of SARS-CoV is tightly integrated with the NTD of the neighboring chain, while that of SARS-CoV-2 is angled close to the cavity of center. Moreover, the HR1 domain of SARS-CoV-2 forms a six-helical bundle structure, which has higher helical stability than SARS-CoV. In addition, there are several reports suggesting that SARS-CoV-2 has a better ability to fuse membrane than SARS-CoV.

### Structure of the SARS-CoV-2 S Protein and Its Binding With ACE2

Recent studies highlight that ACE2 plays a critical role in mediating SARS-CoV-2’s entry. In vitro, the binding affinity of the ACE2 with SARS-CoV-2 RBD is at the nanomolar level, indicating RBD plays important roles in binding (Walls et al., 2020; Tian et al., 2020).

Compared to SARS-CoV, the SARS-CoV-2 RBM has some sequence variations, and the distal end has an obvious change of conformation. Except for that, the structure of the RBD between SARS-CoV-2 and SARS-CoV is similar, and the RMSD on 174 aligned Cα atoms is 1.2 Å (**Figures 7C, D**) (Lan et al., 2020).

The residues of SARS-CoV-2 RBD are essential for binding to ACE2. The RBD of SARS-CoV-2 forms more contacts with the ACE2’s N-terminal helix, the buried area between the RBD and ACE2 is about 878.4Å^2^, whereas, for SARS-CoV, the buried area of RBD and ACE2 is 839.45Å^2^ (**Figure 7G**). In addition, there is a four-residue motif Gly-Val/Gln-Glu/Thr-Ser in SARS-CoV-2, instead of a three-residue motif proline-proline-alanine that is seen in SARS-CoV. Also, the Phe486, located in SARS-CoV-2 RBM, is in different directions and stays in the hydrophobic pockets, which consist of Leu79, Met82, and Tyr83 of ACE2 (Shang et al., 2020; Lan et al., 2020). Taken together, these structural changes in the SARS-CoV-2 RBM enhance the interaction for ACE2 binding.

Furthermore, more forces of interaction were formed in the joint surface between ACE2 and the RBD of SARS-CoV-2 than those in SARS-CoV. There are two virus-binding hotspots on ACE2. One hotspot is ACE2^Lys31^, which forms a salt bridge with Glu35 of the RBD from SARS-CoV-2. Another hotspot is ACE2^Lys353,^ which forms a salt bridge with Asp38 of the RBD from SARS-CoV-2. The Kd value of ACE2 for binding to the RBDs from SARS-CoV or SARS-CoV-2 is 44.2 nM and 185 nM, respectively. In addition, corresponding to the SARS-CoV RBM’s Leu472, Phe486, located in SARS-CoV-2 RBM, has a different orientation to enhance the interactions between RBM and ACE2 (**Figures 7E, F**) (Shang et al., 2020).

In conclusion, the changes of structure from SARS-CoV-2 RBM offer more favorable interactions for binding to ACE2 (Benton et al., 2020; Cao L. et al., 2020; Wu et al., 2020b). According to the known structure of SARS-CoV-2 S protein with ACE2 (Benton et al., 2020; Xu C. et al., 2020), many efforts focus on designing inhibitors to disrupt the interaction between S protein and ACE2. These strategies include competitive binding to the RBD or interference with ACE2 binding.

### Inhibitors of SARS-CoV-2 S Protein

#### Neutralizing Antibodies

Among the inhibitors that prevent the interaction between ACE2 and the RBD, neutralizing antibodies against S protein are the most widely studied. The first reported neutralizing antibody is BD-368-2, which was selected from 14 potent neutralizing antibodies purified from patients’ plasma or blood (Cao Y. et al., 2020; Long et al., 2020). The half-maximal inhibitory concentration (IC_50_) of BD-368-2, against authentic and pseudo SARS-CoV-2, is 15 and 1.2 ng/mL, respectively. BD-368-2 could protect against SARS-CoV-2 *in vivo* experiments, providing valuable therapeutic effects. The second reported neutralizing antibody is 2B04, which could neutralize wild-type SARS-CoV-2 with remarkable potency *in vitro* (IC_50_ less than 2 ng/ml) (Alsoussi et al., 2020). 2B04 reduces the viral load of the lung, prevents systemic transmission in a murine model of SARS-CoV-2 infection, and protects challenged animals from weight loss. Subsequently, three RBD-specific monoclonal neutralizing antibodies, P2C-1F11, P2C-1A3, and P2B-2F6, from a single B cell were reported (Ju et al., 2020). Their IC_50_ are 0.03, 0.28 and 0.41 μg/mL, respectively. These monoclonal antibodies can compete with ACE2 for RBD binding, exhibiting robust anti-SARS-CoV-2 neutralizing activity. Thereafter, a series of REGN neutralizing antibodies REGN10933 and REGN10987 showed high clinically therapeutic effects, with IC_50_ of 42.8 and 40.8 pM, respectively (Baum et al., 2020).

##### Structure of BD23-Fab in Complex With S Trimer

**Figure 8:**
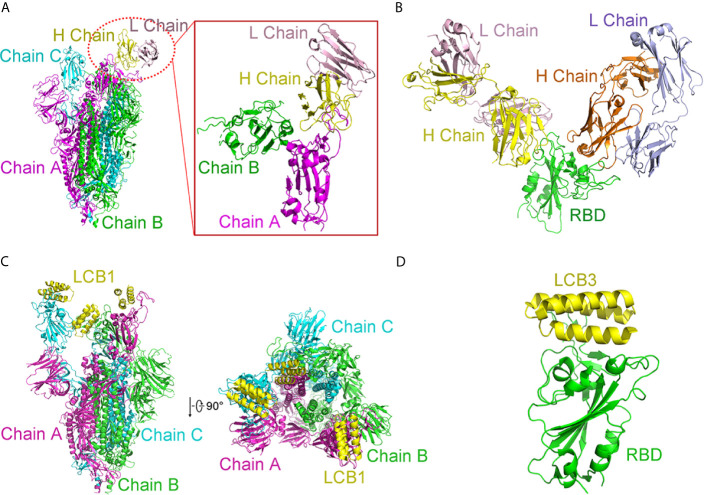
Complex structures of SARS-CoV-2 with antibodies. **(A)** Cryo-EM structure of BD-23 Fab in complex with the spike (S) trimer. The three protomers in the S trimer are shown in green, cyan, and magenta, respectively. BD23-Fab is colored in yellow (heavy chain) and pink (light chain). The figure in the box is an amplified part of the interaction between BD-23 Fab and Chain **(B) (B)** Structure of REGN10933 and REGN10987 in complex with RBD. The heavy chain of REGN10933 is orange and light chain of REGN10933 is blue. The heavy chain of REGN10987 is yellow and light chain of REGN10987 is pink. RBD is shown in green. **(C)** Cryo-EM structure of LCB1 in complex with the S trimer from side view and top view. The three protomers in the S trimer are shown in green, cyan, and magenta, respectively. LCB1 is colored in yellow. **(D)** Cryo-EM structure of LCB3 in complex with the RBD. The LCB3 is shown in yellow, and RBD is colored in green. All structures are drawn by Pymol.

The cryogenic electron microscopy (cryo-EM)-based structure of BD-23 Fab in complex with the trimer of S protein (s trimer) was resolved at an overall resolution of 3.8 Å (Cao L. et al., 2020). In this 3D reconstruction, a single BD-23 Fab binds the “down” RBD in chain B of S trimer. As for binding to the RBD, the BD-23 Fab’s heavy chain variable domain is involved. In fact, a comparison with the complex structure of RBD-ACE2 shows that BD-23 Fab competes with ACE2 for binding to RBD (**Figure 8A**).

##### Structure of Fab Fragments of REGN10933 and REGN10987 in Complex With RBD

In addition to acquiring antibodies from the blood of patients, many studies have attempted to obtain enough antibodies from humanized mice or other mammals and use cocktails to achieve more efficient treatments. In this way, Regeneron Pharmaceuticals developed several prospective antibodies with IC_50_ at the picomolar level (Baum et al., 2020). The complex ternary’s single-particle cryo-EM with a 3.9 Å resolution displays that fab fragments of REGN10933 and REGN10987 can simultaneously bind distinct regions of the RBD. REGN10933 binds at the RBD’s top. The REGN10987 epitope is located on the RBD side with little overlap with the ACE2 binding site (**Figure 8B**).

#### *De Novo* Protein

Interestingly, D. Baker group designed a series of *de novo* mini proteins to mimic RBD antibodies (Cao Y. et al., 2020). Applying shape and chemical complementarity strategies, they designed high-affinity mini binders that compete with ACE2 binding. Ten mini proteins (AHB1, AHB2, and LCB1-LCB8) could bind to the RBD, which affinities are ranging from 100 pM to 10 nM. These inhibitors could prevent the entry of SARS-CoV-2 into Vero E6 cells with IC_50_ from 35 pM to 35 nM. Among them, LCB1 (56 residues) and LCB3 (64 residues) showed stability and are the most potent inhibitors. Their IC_50_ values are almost six-fold that of the most effective monoclonal antibodies reported so far. Cryo-EM structures determined by the study corresponded to their computational LCB1/RBD or LCB3/RBD models (**Figures 8C, D**). In a single spike protein, three RBDs can be used simultaneously. These *de novo* hyper stable mini binders offer good points for new therapeutics of SARS-CoV-2.

Apart from those mentioned above, numerous neutralizing antibodies for SARS-CoV-2 have sprung up (**Figures S2, S3**). Structural evidence suggests that the stoichiometry of RBD with neutralizing antibodies is different (**Figure S2**). For instance, S309 Fab (Pinto et al., 2020), H104 Fab (Lv et al., 2020), and C105 Fab (Barnes et al., 2020) could bind two or three RBDs of S protein. The state of the RBD differs when it binds to different Fab fragments. S309 Fab binds RBD of S protein in the closed state (**Figures S2A, B**). However, H104 Fab and C105 Fab bind RBD of S protein in the open state (**Figures S2C–F**).

Simultaneously, some structures of neutralizing antibodies with RBD were determined. In these structures, RBD could bind one neutralizing antibody, which includes CR3022 (Yuan et al., 2020b), CC12.1, CC12.3 (Yuan et al., 2020a), and so on (**Figures S2A–H**). RBD also binds two different neutralizing antibodies (**Figures S3I–K**) and nanobody (**Figure S3M**). Here, we summarize inhibitors for S protein or RBD as follows (**Table 1**) (Toelzer et al., 2020).

**Table 1:** Summary of inhibitors for spike protein.

Type of inhibitor	Target of inhibitor	Inhibitor	IC_50_ (Pseudovirus)	IC_50_ (Authentic virus)	Kd	EC_50_	ND_50_	Reference
Chemical compounds	HR1 and HR2	EK1C4	15.8 nM	36.5 nM	-	-	-	(Xia et al., 2020)
	HR2	IPB02	80 nM	-	-	-	-	(Zhu et al., 2020)
	COVID-19	Glycyrrhizic acid	-	Reduces IC_50_ by about 10-fold	-	-	-	(Bailly and Vergoten, 2020)
	ACE2	Captopril	-	-	-	-	-	(Biyani et al., 2020)
		Enalapril	-	-	-	-	-	(Sharif-Askari et al., 2020)
		ARB	-	-	-	-	-	(Chung et al., 2020)
		Lisinopril	-	-	-	-	-	(Khelfaoui et al., 2020)
Neutralizing antibodies	Spike	BD-217	0.031 μg/ml	0.84 μg/ml	0.29 nM	-	-	(Cao Y. et al., 2020)
		BD-218	0.011 μg/ml	0.29 μg/ml	0.039 nM	-	-	(Cao Y. et al., 2020)
		BD-236	0.037 μg/ml	0.52 μg/ml	2.8 nM	-	-	(Cao Y. et al., 2020)
		BD-361	0.020 μg/ml	0.780 μg/ml	1.3 nM	-	-	(Cao Y. et al., 2020)
		BD-368	0.035 μg/ml	1.600 μg/ml	1.2 nM	-	-	(Cao Y. et al., 2020)
		BD-368-2	0.001.2 μg/ml	0.015 μg/ml	0.82 nM	-	-	(Cao Y. et al., 2020)
		BD-395	0.020 μg/ml	0.270 μg/ml	0.36 nM	-	-	(Cao Y. et al., 2020)
		2B04	1.46 ng/ml	-	-	-	-	(Alsoussi et al., 2020)
		CA1	-	-	4.68 ± 1.64 nM	-	0.38 μg/ml	(Shi et al., 2020)
		CB6	-	-	2.49 ± 1.65 nM	-	0.036 ± 0.007 μg/ml	(Shi et al., 2020)
		P2C-1F11	0.03 μg/ml	0.03 μg/ml	2.12 nM	-	-	(Ju et al., 2020)
		P2B-2F6	0.05 μg/ml	0.41 μg/ml	5.14 nM	-	-	(Ju et al., 2020)
		P2C-1A3	0.62 μg/ml	0.28 μg/ml	2.47 nM	-	-	(Ju et al., 2020)
		S309 Fab	-	79 ng/ml	0.81 nM	-	-	(Pinto et al., 2020)
		H11-H4-Fc	61 nM	-	5.5 nM	-	6 nM	(Huo et al., 2020)
		H11-D4-Fc	161 nM	-	10 nM	-	18 nM	(Huo et al., 2020)
		VHH72-Fc	262 nM	-	40nM	-	0.2 μg/ ml	(Huo et al., 2020)
		CR3022 Fab	347 nM	-	115 nM	-	-	(Yuan et al., 2020a)
		CR3022 IgG	-	-	1 nM	-	-	(Yuan et al., 2020a)
		H014 Fab	3 nM	38 nM	0.096 nM	0.7 nM	-	(Lv et al., 2020)
		REGN10933	42.8 pM	37.4 pM	3.37 nM	5.79 pM	-	(Baum et al., 2020)
		REGN10987	40.6 pM	42.1 pM	45.2 nM	6.33 pM	-	(Baum et al., 2020)
		REGN10989	7.23 pM	7.38 pM	3.65 nM	4.86 pM	-	(Baum et al., 2020)
		REGN10934	54.4 pM	28.3 pM	4.86 nM	2.72 pM	-	(Baum et al., 2020)
		C105 Fab	26.1 ng/mL	-	-	-	-	(Barnes et al., 2020)
		CV30	0.03 μg/mL	-	3.6 nM	-	-	(Hurlburt et al., 2020)
		EY6A	54 nM	-	2 nM	-	0.5 nM	(Zhou et al., 2020)
		CC12.1Fab	20 ng/ml	20 ng/ml	17 nM	-	-	(Yuan et al., 2020a)
		CC12.3 Fab	20 ng/ml	20 ng/ml	14 nM	-	-	(Yuan et al., 2020a)
		COVA2-39	0.036 µg/ml	0.054 µg/ml	21 nM	-	-	(Wu N. C. et al., 2020)
		COVA2-04	0.22 µg/ml	2.5 µg/ml	40 nM	-	-	(Wu N. C. et al., 2020)
		4A8	-	0.39 μg/ml	92.7 nM	0.61μg/ml	-	(Chi et al., 2020)
Nanobody		Ty1	54 nM	-	5–10 nM	-	-	(Hanke et al., 2020)

IC_50_, half maximal inhibitory concentration; Kd, dissociation constant; EC_50_, concentration for half of maximal effect; ND_50_, half neutralizing dose.

#### Chemical Compounds

There are no effective drugs to prevent the SARS-CoV-2 spread. Thus, therapeutic agents to treat infected individuals are urgently required. Membrane fusion is a crucial step in viral infection. The previously developed coronavirus fusion inhibitors have been shown to prevent virus fusion by targeting the S2 subunit. A pan-coronavirus fusion inhibitor EK1, targeting the subunit S2’s HR1 domain, could inhibit infection of SARS-CoV and MERS-CoV. Subsequently, an EK1 derivative, EK1C4, was found to inhibit the membrane fusion and pseudo-viral infection of SARS-CoV-2 with an IC_50_ of 1.3 nM and 15.8 nM, respectively (Xia et al., 2020). In addition, IPB02, a lipopeptide fusion inhibitor based on HR2 sequence, showed efficient antiviral activity (Ling et al., 2020; Zhu et al., 2020).

### Inhibitors of ACE2

Besides inhibiting the S protein, blocking ACE2 may also be a potential strategy to inhibit the coronavirus infection. Some researchers hope to interrupt the interaction between ACE2 and SARS-CoV-2 by employing existing FDA-approved ACE2 inhibitors (including captopril, enalapril, and lisinopril) (Vaduganathan et al., 2020). In fact, the application of ACE2 inhibitors brought two contradictory impacts on the patients. In some cases, ACE2 inhibitors could reduce inflammation. While in other cases, the inhibitors would improve the entry of viruses. Actually, these ACE2 inhibitors mainly act on the peptidase domain of ACE2, not on the interface for SARS-CoV-2 binding. Moreover, some adverse effects were reported, such as hypertension, heart failure, and these ACE2 inhibitors cause serious side effects in diabetic patients (Bavishi et al., 2020). Therefore, existing FDA-approved ACE2 inhibitors are not an appropriate option for treating SARS-CoV-2 patients (South et al., 2020).

Apart from that, chloroquine (CQ) and hydroxychloroquine (HCQ) were found to be potential therapeutic agents for COVID-19 (Xiu et al., 2020). CQ is a 4- aminoquinoline compound derived from quinine, which has been used to treat malaria, amoebiasis, and SARS-CoV. Studies demonstrated that CQ could increase the endosomal pH value to one which is higher than that required for cell fusion. In addition, CQ could damage the terminal glycosylation of ACE2 receptors, decreasing the interaction between viruses and their ACE2 receptor. Under cell culture conditions, CQ can prevent the interaction between the ACE2 and SARS-CoV RBD, for which ED_50_ value is 4.4 μM (Vincent et al., 2005). CQ could inhibit SARS-CoV-2, which IC_50_ value is 1.13 μM (Wang M. L. et al., 2020). HCQ is an analogue of CQ, in which one of the N-ethyl substituents of CQ is β-hydroxylated. Hydroxychloroquine has the same mechanism as CQ but shows more tolerability than CQ (Rainsford et al., 2015). The results showed that the IC_50_ value of hydroxychloroquine is 0.72 μM after 48 h of incubation (Yao X. et al., 2020).

Even though CQ and HCQ show good antiviral activity, safety, and effectiveness, studies found that they may cause QT prolongation (Stas et al., 2008), ventricular arrhythmias (Chen et al., 2006), retinopathy (Yaylali et al., 2013), and other heart-related toxicities which may be harmful to severely ill patients. Overall, the treatment with CQ and HCQ has certain limitations.

## Non-Structural Protein and Its Inhibitors

### Structure of Mpro and Its Inhibitors

The main proteinase (Mpro or 3C-like protease) is a cysteine proteinase with a serine proteinase-like structure that can cleave the main part of the polyprotein at 11 conserved sites, releasing RdRp, helicase, and other proteins for viral RNA replication. The functional role of Mpro for the maturation of viral proteins, coupled with the human lack of closely related homology, suggests that Mpro is an attractive target designing antiviral drugs (Ziebuhr et al., 2000; Ziebuhr, 2005; Yang et al., 2005).

#### Structure of Mpro With N3

**Figure 9:**
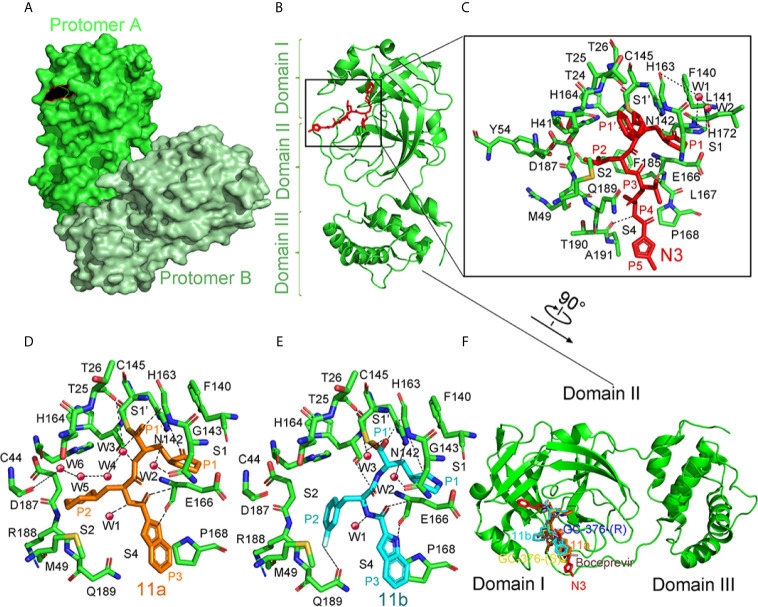
The crystal structure of SARS-COV-2 virus Mpro in complex with inhibitors. **(A)** Surface representation of the homodimer of Mpro. Protomer A is in green, and protomer B is in pale green. **(B)** Cartoon representation of one protomer of the dimeric Mpro-inhibitor complex. The protomer is in green. N3 is in red sticks. **(C)** A zoomed view for the substrate-binding pocket. The key residues, which form the binding pocket, are shown as green sticks, the two waters, assigned as W1 and W2, are shown as red spheres. Four subsites, S1′, S1, S2, and S4, are labeled. P1, P1′, P2, P3, P4, and P5 sites of N3 are shown. Hydrogen bonds are indicated as dashed lines. **(D)** Close-up view of the 11a binding pocket. The residues involved in inhibitor binding are shown as green sticks. 11a and water molecules are shown as orange sticks and red spheres, respectively. Four subsites, S1′, S1, S2, and S4, are labeled. P1, P1′, P2, and P3 sites of 11a are indicated. Hydrogen bonds are indicated as dashed lines. **(E)** Close-up view of the 11b binding pocket. 11b and water molecules are shown as cyan sticks and red spheres, respectively. Four subsites, S1′, S1, S2, and S4, are labeled. P1, P1′, P2, and P3 sites of 11b are indicated. Hydrogen bonds are indicated as dashed lines. **(F)** Comparison of the inhibitor binding modes in SARS-CoV-2 Mpro. Two configurations (S and R) of GC-376, 11a, 11b, N3, and Boceprevir are shown in yellow, blue, orange, cyan, red, and dirty violet, respectively. All structures are drawn by Pymol.

A Michael acceptor-based peptidomimetic inhibitor N3 designed by computer-aided drug design (CADD) can inhibit Mpro proteases in MERS-CoV and SARS-CoV (Xue et al., 2008). Consistently, N3 could also fit well inside the SARS-CoV-2’s substrate-binding pocket (Jin et al., 2020). In the structure, two Mpro form dimers *via* 2-fold symmetry axes of crystallography (named protomer A and B) (**Figure 9A**). Each protomer is formed by three domains (**Figure 9B**). Antiparallel β-barrel structures are shown in domain I (residues 8–101) and domain II (residues 102–184). Domain III (residues 201–303) consists of 5 α-helices. Mpro has a Cys–His catalytic dyad, and there is a cleft between domain I and II as the N3-binding site.

The electron density indicates that the Cb of the vinyl group on N3 and the Mpro catalytic sites Cys145 form a standard 1.8 Å C–S covalent bond, confirming that the Michael addition has occurred (**Figure 9C**). Five parts from N3 inserts Mpro active sites as P1, P2, P3, P4, and P5 (Wang et al., 2017). The lactam, located on P1, inserts into the S1 subsite, forming a hydrogen bond with His163 in protomer A. At the P2 site, the Leu’s side chain inserts into the hydrophobic S2 subsite. Val at P3’s side chain is solvent-exposed. The P4 side Ala is encircled by Leu167’s and Phe185’s side chains, and the main chain of Glu189, all of which form a small hydrophobic pocket. At P5, there are van der Waals contacting with Pro168. Through plaque-reduction assay, N3 showed an inhibitory effect on SARS-CoV-2, with individual half-maximal effective concentration (EC_50_) values of 16.77 μM (Jin et al., 2020).

#### Structure of Mpro With 11a and 11b

By analyzing the structure-activity relationship (SAR) of the reported inhibitor and substrate binding pocket, two compounds 11a and 11b with more effective inhibition than N3 were designed to target SARS-CoV-2 Mpro (Dai et al., 2020).

The electron density map in the complex structure clearly shows the extended conformation of compound 11a in the Mpro substrate-binding pocket (**Figure 9D**). The Mpro catalytic sites Cys145 forms a covalent bond (1.8-Å) with the aldehyde-based C of 11a (**Figure 9E**). At P1, the (S)-g-lactam ring of 11a fits well into the S1 site. At P3, the 11a indolyl group is exposed to the S4 position, and Glu166 could stabilize by a hydrogen bond. When binding 11a, multiple water molecules, named W1 to W6, play important roles. Through a 2.9 Å hydrogen bond, W1 has interaction with the amide bond of 11a. As for W2 to W6, the five molecules stabilize 11a in the binding pocket. The 11b shows a similar inhibition binding mode with 11a. (**Figure 9F**). The biggest difference in binding mode is at P2. The 3-fluorophenyl group in 11b has a downward rotation of about 42.7 degrees relative to the cyclohexyl group in 11a (Dai et al., 2020). The binding modes of 11 a and 11 b in the Mpro complex structure are similar to the previously reported N3 and other inhibitors (**Figure 9F**). More water molecules are involved in binding hydrogen bonds in 11 a and 11 b, and the functional groups on the P2 are involved in some additional interactions, which may be the reason for their enhanced inhibition (Wang et al., 2016; Zhang et al., 2020). Here we summarize inhibitors for Mpro as follows (**Table 2**).

**Table 2:** The summary of recently reported Mpro inhibitors.

Compounds	SARS-CoV-2	SARS-CoV-2	Development Stage
	Mpro inhibition (μM)	Antiviral activity (μM)	
N3	Kobs/[I]=11,300 ± 800M^-1^S^-1^	EC_50 =_ 16.77 ± 1.7	Preclinical; not tested in animals models
Ebselen	IC_50 =_ 0.67 ± 0.09	EC_50 =_ 4.67 ± 0.80	In clinical trials
11a	IC_50 =_ 0.053 ± 0.005	EC_50 =_ 0.53 ± 0.01	Preclinical; Favorable PK in rats and low toxicity in rats and dogs
11b	IC_50 =_ 0.040 ± 0.002	EC_50 =_ 0.72 ± 0.09	Preclinical; Favorable PK in rats
Boceprevir	IC_50 =_ 4.13 ± 0.61 Ki=1.18	EC_50 =_ 1.31 ± 0.58	FDA-approved
			HCV drug
GC-376	IC_50 =_ 0.03 ± 0.008	EC_50 =_ 3.37 ± 1.68	Preclinical; Tested in felines
	K2/KI=40,800M^-1^S^-1^		
MG-132	IC_50 =_ 3.90 ± 1.01	NT.	Preclinical; Tested in mice
Calpain inhibitor II	IC_50 =_ 0.97 ± 0.27	EC_50 =_ 2.07 ± 0.76	Preclinical; not tested in animals models
	Ki=0.40		
Calpain inhibitor XII	IC_50 =_ 0.45 ± 0.06	EC_50 =_ 0.49 ± 0.18	Preclinical; not tested in animals models
	Ki=0.13		

#### Mpro Inhibitors From FDA Approved Library

For a medical emergency, scientists began to re-screen drugs from all protease inhibitors approved by the FDA, which could inhibit Mpro. The established FRET assay was used to identify potential SARS-CoV-2 Mpro inhibitors by screening all protease inhibitors from the Selleckchem bioactive compound library (Ma et al., 2020).

Noticeable findings are as follows: (1) Boceprevir, an FDA-approved HCV drug, inhibits the Mpro’s enzymatic activity with IC_50_ of 4.13 µM and EC_50_ of 1.90 µM against the SARS-CoV-2 virus in the cellular viral cytopathogenic effect (CPE) assay. (Ma et al., 2020). (2) GC-376, an investigational veterinary drug that has the highest enzymatic inhibition against the Mpro with an IC_50_ value of 0.03 µM (Kim et al., 2016; Pedersen et al., 2018) among candidate inhibitors so far. It showed promising activity against the SARS-CoV-2 virus (EC_50_ = 3.37 µM). GC-376 can use two different configurations R and S which may explain its high binding affinity to the target Mpro. (3) Calpain/cathepsin inhibitors. Calpain inhibitors II and XII, MG-132, are three potent inhibitors of Mpro with single-digit submicromolar efficacy in the enzymatic assay. Calpain inhibitors II and XII inhibit SARS-CoV-2 with EC_50_ values of 2.07 µM and 0.49 µM, respectively. MG-132 inhibits SARS-CoV-2 with IC_50_ values of 3.90 µM (Barnard et al., 2004).

In summary, different potent SARS-CoV-2 Mpro inhibitors had been found with potent antiviral activity. Based on these hits, further development may lead to clinical application towards SARS-CoV-2 infections.

### Structure of RdRp and Its Inhibitors

Remdesivir is an adenosine analogue that converts intracellularly into active drugs in the form of triphosphate (RTP), which binds to the nascent viral RNA chain, leading to premature termination (Velthuis et al., 2011; Ahn et al., 2012; Subissi et al., 2014; Kirchdoerfer and Ward, 2019; Wang M. L. et al., 2020). (including SARS/MERS/CoV/Ebola) Remdesivir is considered to be a promising antiviral drug against various RNA viruses (including SARS/MERS/Ebola virus) (Mulangu et al., 2019). Evidence showed that Remdesivir also efficiently inhibited SARS-CoV-2 infection through RdRp (van Hemert et al., 2008; Gong and Peersen, 2010; Sheahan et al., 2017).

**Figure 10:**
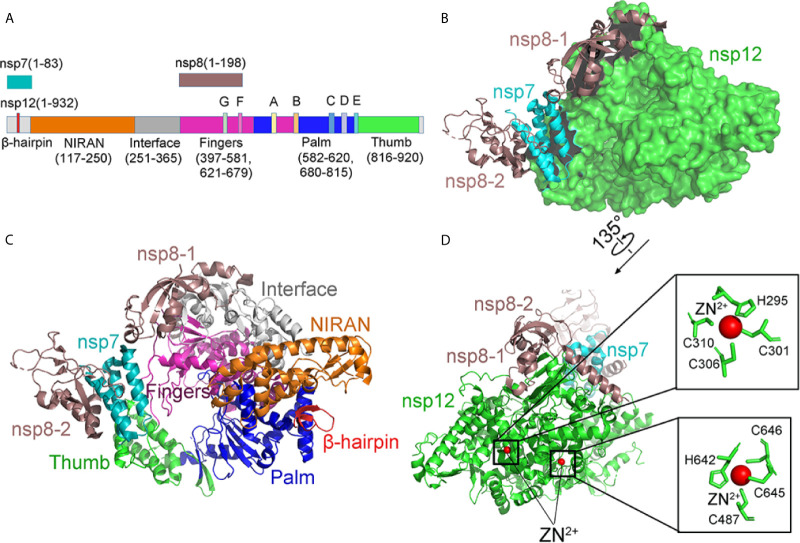
The structure of the apo RdRp complex. **(A)** The schematic diagram for the components of the RdRp complex, which contains nsp7, nsp8, and nsp12. The polymerase motif (A-G) and β-hairpin are highlighted because it is unique to the SARS-CoV-2. **(B, C)** Two views structure of the apo nsp12-nsp7-nsp8 complex. nsp12 consists of an N-terminal β-hairpin (residues 31–50) and an extended nidovirus RdRp-associated nucleotidyl-transferase domain (NiRAN, residues 115–250). An interface domain (residues 251–365) is following the NiRAN domain. The nsp12 RdRp domain shows the canonical cupped right-handed configuration with the finger subdomain. NiRAN, Interface, Fingers, Thumb, Palm, nsp7, nsp8 are shown in orange, gray, hot pink, green, blue, cyan, and violet, respectively. **(D)** The conserved zinc-binding motifs in the apo structure rendered in ribbon are highlighted. The residues are shown as green sticks, and zinc is shown as red spheres. All structures are drawn by Pymol.

Cryo-EM structures of RdRp provide insights into the inhibition mechanism of remdesivir (Yin et al., 2020) (**Figures 10B, C**). Different from the SARS-CoV RdRp, nsp12 of SARS-CoV-2 RdRp has an additional β-hairpin at the N-terminus (residues 31–50), as well as an extended nidovirus RdRp-associated nucleotidyl-transferase domain, which has three β-strands and seven helices (NiRAN, residues 115–250) (Lehmann et al., 2015; Gao et al., 2020). An interface domain (residues 251–365) is near the NiRAN domain (**Figures 10A, C**). The nsp12 RdRp domain displays the finger subdomain (resides 397–581 and residues 621–679), forming a closed circle with the thumb subdomain (residues 819–920). The closed conformation is stabilized by a combination of an nsp7 and an nsp8. Two zinc ions were found in the H295-C301-C306-C310 and C487-H642-C645-C646 conserved metal-binding motifs (**Figure 10D**). These zinc ions may act as conserved structural components to maintain RdRP structural integrity (Yin et al., 2020).

**Figure 11:**
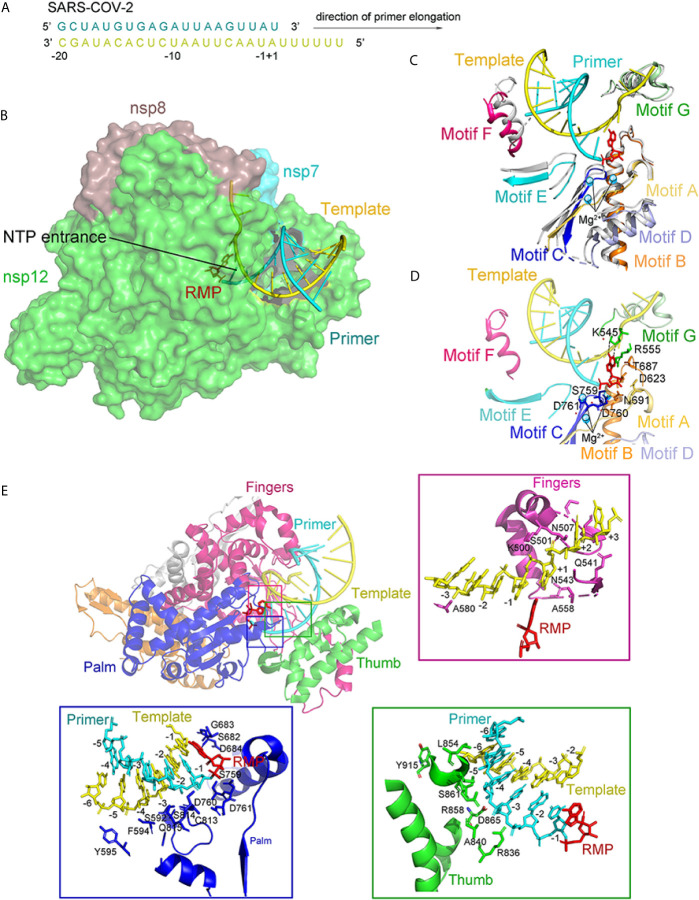
The structure of the template-RTP RdRp complex. **(A)** The sequence of the RNA duplex with a 5′ U10 overhang as a template for extending primer and assembling the RdRp-RNA complex. **(B)** Cryo-EM Structure of the Remdesivir and RNA bound RdRp complex. Template and Primer are shown as yellow and cyan. **(C)** The conserved RdRp motifs (A to G) of the RNA-bound complex overlap with the apo structure, and color in gray, with a close view at the active site. Motifs (A to G) are shown in light orange, orange, blue, light blue, aquamarine, warm pink, and pale green, respectively. **(D)** A close view of the RdRp active site, showing the covalently bound RMP, magnesium ions, and pyrophosphate. Key residues and bases interacting with Remdesivir are shown. **(E)** Remdesivir-bound RdRp complex and protein-RNA interactions in the RNA. All structures are drawn by Pymol.

The structure of the template-RTP RdRp complex shows 11-base RNA in the primer strand, 14-base RNA in the template strand, and remdesivir in its monophosphate form (RMP), which is covalently linked to the primer strand (**Figures 11A–C**). Most protein–RNA interactions involve RNA ribose phosphate backbone directly linked to the 2′-OH groups, which provides the basis for distinguishing RNA from DNA. There are no base pairs in RNA from nsp12 to template-primer, indicating that the RdRP was independent of the RNA sequence. It is consistent with the fact that RdRp enzyme activity does not require specific sequences during the elongation step. At the 3′ end of the primer strand is RMP, covalently bound to the primer strand at the +1 position. Additional nucleotides at the +2 and +3 positions of the template strand interact with residues at the back of the finger subdomain (**Figure 11E**). As observed in the structure, only one RMP was assembled into the primer strand though the presence of excess RTP in a complex assembly (Tchesnokov et al., 2019; Gordon et al., 2020).

The RMP position is at the center of the active catalytic site (**Figure 11D**). RMP interacts with the upstream bases from the primer chains to form base stacking and forms two hydrogen bonds with the uridine groups from the template chains. In addition, RMP interacts with the side chains of K545 and R555. There were two magnesium ions near the bound RMP. Both magnesium ions interact with the diester phosphate backbone to form a part of the active catalytic site (**Figure 11D**).

Seven conserved motifs construct the active catalytic site of the nsp12 RdRp from A to G (**Figures 10C** and **11C**). Motifs ABCD are from the palm subdomain forming the active catalytic center (**Figure 11C**). Regarding the two magnesium ions in the catalytic center, both D760 and D761 are involved in the coordination (**Figure 11D**) (te Velthuis, 2014).

Interesting differences between the apo and complex structures were revealed by structural comparison (**Figure 11C**). Firstly, nsp7 moved toward the RdRp, resulting in the interface’s rearrangement and lead to a weaker association of the second nsp8 in the complex. Secondly, the loop, connecting the first and second helix of the thumb subdomain, moved outward to adapt the binding of the double-stranded RNA helix. Thirdly, residues K500 and S501 from motif G moved to accommodate the template strand RNA’s binding. Except for these changes, nsp12 in the apo and the RNA-complex are very similar (Venkataraman et al., 2018). In particular, the structural elements of the active catalytic site can be accurately superimposed, suggesting that RdRp is a relatively stable enzyme that can function as a replicase.

Besides remdesivir, several nucleotides, including galidesivir, ribavirin, favipiravir, and EIDD-2801, inhibit SARS-CoV-2 replication efficiently (Gordon et al., 2020). Especially, EIDD-2801 showed 3 to 10 folds more effective than remdesivir in preventing SARS-CoV-2 replication.

## Discussion

As the third widespread coronavirus that threatens human beings, SARS-CoV-2 has brought great disaster to the world (Yao et al., 2020a; Liang et al., 2020). Unfortunately, the SARS-CoV-2 variant D614G is more infectious than the ancestral SARS-CoV-2 (Plante et al., 2021). The analysis shows that the destruction of the interaction between D614 and T859 and the increase in the proportion of open RBD may be the reason for the increase in SARS-CoV-2 infection. Recently, other mutations, such as E484Q and L452R, have emerged and have decreased the effect of the vaccine. Fortunately, some patients who contracted SARS-CoV-2 recovered quickly after receiving cocktail therapy (Baum et al., 2020). The cocktail’s formula includes Regeneron’s REGN10933 and REGN10987 antibodies, zinc, vitamin D, famotidine, melatonin, and aspirin. Current drug candidates mainly inhibit S protein and some non-structural proteins (Mpro, PLpro, helicase, and RdRp). Small molecule inhibitors and neutralizing antibodies are potential therapies for SARS-CoV-2 infection.

For small molecule inhibitors, some FDA-approved compounds for clinical use have shown efficacy against SARS-CoV-2. Remdesivir, an inhibitor developed for the Ebola virus, inhibits SARS-CoV-2 by binding RNA replicase RdRp. Compared to small molecules, the neutralizing antibodies exhibit robust inhibition against SARS-CoV-2 infection. The neutralizing antibodies BD-368-2 (Cao L. et al., 2020), BD23-Fab (Cao L. et al., 2020), and P2B-2F6 (Ju et al., 2020) against S protein showed good IC_50_ values and could effectively compete with ACE2 binding. The IC_50_ values of neutralizing antibodies REGN10933 and REGN10987 can be as low as a few picomoles, which play an essential role in successful cocktail therapy. Besides this antibody cocktail, two *de novo* mini proteins designed by CADD with high affinity to S protein, LCB1and LCB3, show effective inhibition to the invasion of SARS-CoV-2 to host cells (Cao L. et al., 2020). The structure of the neutralizing antibodies and SARS-CoV-2 S protein complex provides a robust basis, further promoting the understanding of antibody-based therapy. In fact, many patients have saved their lives based on the application of antibodies.

To date, human beings have experienced several epidemic outbreaks, and each episode has had varying degrees of negative impacts on health, economy, psychology, and human behavior. When it comes to coronavirus, researchers have worked hard to discover its replication and pathogenesis and have gained some achievements in this regard. As we all know, many viruses have been present in natural reservoirs for a long time. Owing to human activities, viruses spread from natural hosts to humans or other animals. Unfortunately, people still cannot predict when the virus will arrive and what impact the virus will have. In this review, we provide the origin and evolution of SARS-CoV-2 to show that people should maintain the barrier between natural reservoirs and human society. After this worldwide epidemic, finding effective drugs or inhibitors is imminent. Although antibodies have shown positive effects, the defects are still obvious and not easy to overcome. In particular, it is impossible and costly to extract enough neutralizing antibodies from convalescent patients for countless clinical treatments. In this review, we summarize almost all the existing neutralizing antibodies or drugs and display their structures. Comparing or incorporating their characteristics is important to optimize or design more effective drugs. In the future, *de novo* recombinant protein may overcome the bottleneck of antibodies, improving the binding affinity and yield. It is theoretically feasible to minimize the effective antibodies or design *de novo* antibodies based on the structural data. In the case of Ebola, zMapp, consisting of three chimeric MBS, was successfully used to cure two Ebola patients in 2014 (Lyon et al., 2014). Therefore, the commercial antibody will bring hope to SARS-CoV-2 patients.

In addition to the above two treatments, the vaccine’s clinical application will effectively prevent the spread of SARS-CoV-2 (Silveira et al., 2020). For emergency use, the whole virus inactivated vaccine and mRNA vaccine (Park et al., 2020) have shown the prospect of prevention (Calina et al., 2020). In the future, more in-depth research on SARS-CoV-2 will bring new therapeutic schemes for its prevention and treatment.

## Author Contributions

SN and BY were the first authors with equal contributions, and YW and FW were the corresponding authors with equal contributions. All authors contributed to the article and approved the submitted version.

## Funding

The work was supported by National Natural Science Foundation of China (No.31770827 and 21736002), Youth Project of Beijing Natural Science Foundation 5214027, the National Key Research and Development Program of China 2016YFC0906000, Beijing Institute of Technology Innovative Talents Science and Technology Grant 3160012211907, Beijing Institute of Technology Young Backbone Teacher Start-up Fund 3160012221905, the Beijing Institute of Technology Research Fund Program for Young Scholars.

## Conflict of Interest

The authors declare that the research was conducted in the absence of any commercial or financial relationships that could be construed as a potential conflict of interest.

## References

[B1] Ahn D. G. Choi J. K. Taylor D. R. Oh J. W. (2012). Biochemical Characterization of a Recombinant SARS Coronavirus Nsp12 RNA-dependent RNA Polymerase Capable of Copying Viral RNA Templates. Arch. Virol. 157, 2095–2104. 10.1007/s00705-012-1404-x 22791111 PMC7086750

[B2] Alsoussi W. B. Turner J. S. Case J. B. Zhao H. Schmitz A. J. Zhou J. Q.. (2020). A Potently Neutralizing Antibody Protects Mice Against SARS-CoV-2 Infection. J. Immunol. 205, 915–922. 10.4049/jimmunol.2000583 32591393 PMC7566074

[B3] Andersen K. G. Rambaut A. Ian Lipkin W. Holmes E. C. Garry R. F. (2020). The Proximal Origin of SARS-Cov-2. Nat. Med. 26, 450–452. 10.1038/s41591-020-0820-9 32284615 PMC7095063

[B4] Bailly C. Vergoten G. (2020). Glycyrrhizin: An Alternative Drug for the Treatment of COVID-19 Infection and the Associated Respiratory Syndrome? Pharmacol. Ther. 214, 1–16. 10.1016/j.pharmthera.2020.107618 PMC7311916 32592716

[B5] Barnard D. L. Hubbard V. D. Burton J. Smee D. F. Morrey J. D. Otto M. J.. (2004). Inhibition of Severe Acute Respiratory Syndrome-Associated Coronavirus (SarscoV) by Calpain Inhibitors and Beta-D-N4-Hydroxycytidine. Antivir. Chem. Chemother. 15, 15–22. 10.1177/095632020401500102 15074711

[B6] Barnes C. O. West A. P. Jr. Huey-Tubman K. E. Hoffmann M. A. G. Sharaf N. G. Hoffman P. R.. (2020). Structures of Human Antibodies Bound to SARS-CoV-2 Spike Reveal Common Epitopes and Recurrent Features of Antibodies. Cell 182, 828–842. 10.1016/j.cell.2020.06.025 32645326 PMC7311918

[B7] Baum A. Ajithdoss D. Copin R. Zhou A. Lanza K. Negron N.. (2020). Regn-COV2 Antibodies Prevent and Treat SARS-CoV-2 Infection in Rhesus Macaques and Hamsters. Science 370, 1110–1115. 10.1126/science.abe2402 33037066 PMC7857396

[B8] Bavishi C. Maddox T. M. Messerli F. H. (2020). Coronavirus Disease 2019 (Covid-19) Infection and Renin Angiotensin System Blockers. JAMA Cardiol. 5, 745–747. 10.1001/jamacardio.2020.1282 32242890

[B9] Belouzard S. Chu V. C. Whittaker G. R. (2009). Activation of the SARS Coronavirus Spike Protein Via Sequential Proteolytic Cleavage at Two Distinct Sites. P Natl. Acad. Sci. U.S.A. 106, 5871–5876. 10.1073/pnas.0809524106 PMC2660061 19321428

[B10] Benton D. J. Wrobel A. G. Xu P. Roustan C. Martin S. R. Rosenthal P. B.. (2020). Receptor Binding and Priming of the Spike Protein of SARS-CoV-2 for Membrane Fusion. Nature 588, 327–330. 10.1038/s41586-020-2772-0 32942285 PMC7116727

[B11] Biyani C. S. Palit V. Daga S. (2020). The Use of Captopril-Angiotensin Converting Enzyme (Ace) Inhibitor for Cystinuria During COVID-19 Pandemic. Urology 141, 182–183. 10.1016/j.urology.2020.04.057 32333991 PMC7174975

[B12] Bleibtreu A. Bertine M. Bertin C. Houhou-Fidouh N. Visseaux B. (2020). Focus on Middle East Respiratory Syndrome Coronavirus (MERS-Cov). Med. Maladies Infect. 50, 243–251. 10.1016/j.medmal.2019.10.004 PMC7125975 31727466

[B13] Bosch B. J. van der Zee R. de Haan C. A. M. Rottier P. J. M. (2003). The Coronavirus Spike Protein is a Class I Virus Fusion Protein: Structural and Functional Characterization of the Fusion Core Complex. J. Virol. 77, 8801–8811. 10.1128/Jvi.77.16.8801-8811.2003 12885899 PMC167208

[B14] Burkard C. Verheije M. H. Wicht O. van Kasteren S. I. van Kuppeveld F. J. Haagmans B. L.. (2014). Coronavirus Cell Entry Occurs Through the Endo-/Lysosomal Pathway in a Proteolysis-Dependent Manner. PloS Pathog. 10, 1–17. 10.1371/journal.ppat.1004502 PMC4223067 25375324

[B15] Calina D. Docea A. O. Petrakis D. Egorov A. M. Ishmukhametov A. A. Gabibov A. G.. (2020). Towards Effective COVID-19 Vaccines: Updates, Perspectives and Challenges (Review). Int. J. Mol. Med. 46, 3–16. 10.3892/ijmm.2020.4596 32377694 PMC7255458

[B16] Cao L. Goreshnik I. Coventry B. Case J. B. Miller L. Kozodoy L.. (2020). De Novo Design of Picomolar SARS-CoV-2 Miniprotein Inhibitors. Science 370, 426–431. 10.1126/science.abd9909 32907861 PMC7857403

[B17] Cao Y. Su B. Guo X. Sun W. Deng Y. Bao L.. (2020). Potent Neutralizing Antibodies Against SARS-CoV-2 Identified by High-Throughput Single-Cell Sequencing of Convalescent Patients’ B Cells. Cell 182, 73–84. 10.1016/j.cell.2020.05.025 32425270 PMC7231725

[B18] Cao S. Wang H. H. Luhur A. Wong S. M. (2005). Yeast Expression and Characterization of SARS-Cov N Protein. J. Virol. Methods 130, 83–88. 10.1016/j.jviromet.2005.06.010 16026862 PMC7112830

[B19] Cauchemez S. Van Kerkhove M. D. Riley S. Donnelly C. A. Fraser C. Ferguson N. M. (2013). Transmission Scenarios for Middle East Respiratory Syndrome Coronavirus (Mers-CoV) and How to Tell Them Apart. Eurosurveillance 18, 7–13. 10.1186/1475-2875-12-204 PMC4088931 23787162

[B20] Chafekar A. Fielding B. C. (2018). MERS-Cov: Understanding the Latest Human Coronavirus Threat. Viruses-Basel 10, 1–22. 10.3390/v10020093 PMC5850400 29495250

[B21] Chang C. K. Hou M. H. Chang C. F. Hsiao C. D. Huang T. H. (2014). The SARS Coronavirus Nucleocapsid Protein - Forms and Functions. Antivir. Res. 103, 39–50. 10.1016/j.antiviral.2013.12.009 24418573 PMC7113676

[B22] Chan J. F. W. Lau S. K. P. To K. K. W. Cheng V. C. C. Woo P. C. Y. Yuen K. Y. (2015). Middle East Respiratory Syndrome Coronavirus: Another Zoonotic Betacoronavirus Causing SARS-Like Disease. Clin. Microbiol. Rev. 28, 465–522. 10.1128/Cmr.00102-14 25810418 PMC4402954

[B23] Chen Y. Q. Rajashankar K. R. Yang Y. Agnihothram S. S. Liu C. Lin Y. L.. (2013). Crystal Structure of the Receptor-Binding Domain From Newly Emerged Middle East Respiratory Syndrome Coronavirus. J. Virol. 87, 10777–10783. 10.1128/Jvi.01756-13 23903833 PMC3807420

[B24] Chen B. Tian E.-K. He B. Tian L. Han R. Wang S.. (2020). Overview of Lethal Human Coronaviruses. Signal Transduct. Target. Ther. 5, 89. 10.1038/s41392-020-0190-2 32533062 PMC7289715

[B25] Chen C. Y. Wang F. L. Lin C. C. (2006). Chronic Hydroxychloroquine Use Associated With QT Prolongation and Refractory Ventricular Arrhythmia. Clin. Toxicol. (Phila) 44, 173–175. 10.1080/15563650500514558 16615675

[B26] Chi X. Yan R. Zhang J. Zhang G. Zhang Y. Hao M.. (2020). A Neutralizing Human Antibody Binds to the N-terminal Domain of the Spike Protein of SARS-Cov-2. Science 369, 650–655. 10.1126/science.abc6952 32571838 PMC7319273

[B27] Chung M. K. Karnik S. Saef J. Bergmann C. Barnard J. Lederman M. M.. (2020). Sars-CoV-2 and ACE2: The Biology and Clinical Data Settling the ARB and ACEI Controversy. Ebiomedicine 58, 1–10. 10.1016/j.ebiom.2020.102907 PMC7415847 32771682

[B28] Coronaviridae Study Group of the International Committee on Taxonomy of V. (2020). The Species Severe Acute Respiratory Syndrome-Related Coronavirus: Classifying 2019-nCoV and Naming it SARS-Cov-2. Nat. Microbiol. 5, 536–544. 10.1038/s41564-020-0695-z 32123347 PMC7095448

[B29] Cui J. Li F. Shi Z. L. (2019). Origin and Evolution of Pathogenic Coronaviruses. Nat. Rev. Microbiol. 17, 181–192. 10.1038/s41579-018-0118-9 30531947 PMC7097006

[B30] Dai W. H. Zhang B. Jiang X. M. Su H. X. Li J. A. Zhao Y.. (2020). Structure-Based Design of Antiviral Drug Candidates Targeting the SARS-CoV-2 Main Protease. Science 368, 1331–1335. 10.1126/science.abb4489 32321856 PMC7179937

[B31] Forni D. Cagliani R. Clerici M. Sironi M. (2017). Molecular Evolution of Human Coronavirus Genomes. Trends Microbiol. 25, 35–48. 10.1016/j.tim.2016.09.001 27743750 PMC7111218

[B32] Fouchier R. A. M. Kuiken T. Schutten M. van Amerongen G. van Doornum J. van den Hoogen B. G.. (2003). Aetiology - Koch’s Postulates Fulfilled for SARS Virus. Nature 423, 240–240. 10.1038/423240a 12748632 PMC7095368

[B33] Gao X. M. Xu H. Zhang B. N. Tao T. Liu Y. L. Xu D. J.. (2019). Interaction of N-acetyl-seryl-aspartyl-lysyl-proline With the Angiotensin-Converting Enzyme 2-Angiotensin-(1-7)-Mas Axis Attenuates Pulmonary Fibrosis in Silicotic Rats. Exp. Physiol. 104, 1562–1574. 10.1113/Ep087515 31290182

[B34] Gao Y. Yan L. Huang Y. Liu F. Zhao Y. Cao L.. (2020). Structure of the RNA-dependent RNA Polymerase From COVID-19 Virus. Science 368, 779–782. 10.1126/science.abb7498 32277040 PMC7164392

[B35] Gong P. Peersen O. B. (2010). Structural Basis for Active Site Closure by the Poliovirus RNA-dependent RNA Polymerase. Proc. Natl. Acad. Sci. U.S.A. 10, 22505–22510. 10.1073/pnas.1007626107 PMC3012486 21148772

[B36] Gordon C. J. Tchesnokov E. P. Woolner E. Perry J. K. Feng J. Y. Porter D. P.. (2020). Remdesivir is a Direct-Acting Antiviral That Inhibits RNA-dependent RNA Polymerase From Severe Acute Respiratory Syndrome Coronavirus 2 With High Potency. J. Biol. Chem. 295, 6785–6797. 10.1074/jbc.RA120.013679 32284326 PMC7242698

[B37] Guo Y. Korteweg C. McNutt M. A. Gu J. (2008). Pathogenetic Mechanisms of Severe Acute Respiratory Syndrome. Virus Res. 133, 4–12. 10.1016/j.virusres.2007.01.022 17825937 PMC7114157

[B38] Hamming I. Cooper M. E. Haagmans B. L. Hooper N. M. Korstanje R. Osterhaus A. D.. (2007). The Emerging Role of ACE2 in Physiology and Disease. J. Pathol. 212, 1–11. 10.1002/path.2162 17464936 PMC7167724

[B39] Hanke L. Vidakovics Perez L. Sheward D. J. Das H. Schulte T. Moliner-Morro A.. (2020). An Alpaca Nanobody Neutralizes SARS-CoV-2 by Blocking Receptor Interaction. Nat. Commun. 11, 1–9. 10.1038/s41467-020-18174-5 32887876 PMC7473855

[B40] Hemnes A. R. Rathinasabapathy A. Austin E. A. Brittain E. L. Carrier E. J. Chen X. P.. (2018). A Potential Therapeutic Role for Angiotensin-Converting Enzyme 2 in Human Pulmonary Arterial Hypertension. Eur. Respir. J. 51, 1–20. 10.1183/13993003.02638-2017 PMC6613216 29903860

[B41] Hoffmann M. Kleine-Weber H. Schroeder S. Krüger N. Herrler T. Erichsen S.. (2020). SARS-Cov-2 Cell Entry Depends on ACE2 and TMPRSS2 and Is Blocked by a Clinically Proven Protease Inhibitor. Cell 181, 271–280. 10.1016/j.cell.2020.02.052 32142651 PMC7102627

[B42] Holmes K. V. (2003). SARS-Associated Coronavirus. New Engl. J. Med. 348, 1948–1951. 10.1056/NEJMp030078 12748314

[B43] Huang C. Wang Y. Li X. Ren L. Zhao J. Hu Y.. (2020). Clinical Features of Patients Infected With 2019 Novel Coronavirus in Wuhan, China. Lancet 395, 497–506. 10.1016/S0140-6736(20)30183-5 31986264 PMC7159299

[B44] Huo J. Le Bas A. Ruza R. R. Duyvesteyn H. M. E. Mikolajek H. Malinauskas T.. (2020). Neutralizing Nanobodies Bind SARS-CoV-2 Spike RBD and Block Interaction With ACE2. Nat. Struct. Mol. Biol. 27, 846–854. 10.1038/s41594-020-0469-6 32661423

[B45] Hurlburt N. K. Seydoux E. Wan Y.-H. Edara V. V. Stuart A. B. Feng J.. (2020). Structural Basis for Potent Neutralization of SARS-CoV-2 and Role of Antibody Affinity Maturation. Nat. Commun. 11, 1–7. 10.1038/s41467-020-19231-9 33110068 PMC7591918

[B46] Jiang R. D. Liu M. Q. Chen Y. Shan C. Zhou Y. W. Shen X. R.. (2020). Pathogenesis of SARS-CoV-2 in Transgenic Mice Expressing Human Angiotensin-Converting Enzyme 2. Cell 182, 50–58. 10.1016/j.cell.2020.05.027 32516571 PMC7241398

[B47] Jiang L. W. Wang N. S. Zuo T. Shi X. L. Poon K. M. V. Wu Y. K.. (2014). Potent Neutralization of MERS-CoV by Human Neutralizing Monoclonal Antibodies to the Viral Spike Glycoprotein. Sci. Transl. Med. 6, 234ra59. 10.1126/scitranslmed.3008140 24778414

[B48] Jin Z. M. Du X. Y. Xu Y. C. Deng Y. Q. Liu M. Q. Zhao Y.. (2020). Structure of M-pro From SARS-CoV-2 and Discovery of its Inhibitors. Nature 582, 289–293. 10.1038/s41586-020-2223-y 32272481

[B49] Ju B. Zhang Q. Ge J. W. Wang R. K. Sun J. Ge X. Y.. (2020). Human Neutralizing Antibodies Elicited by SARS-CoV-2 Infection. Nature 584, 115–119. 10.1038/s41586-020-2380-z 32454513

[B50] Khelfaoui H. Harkati D. Saleh B. A. (2020). Molecular Docking, Molecular Dynamics Simulations and Reactivity, Studies on Approved Drugs Library Targeting ACE2 and SARS-CoV-2 Binding With ACE2. J. Biomol. Struct. Dyn. 5, 1–17. 10.1080/07391102.2020.1803967 PMC7484571 32752951

[B51] Kim Y. Liu H. W. Kankanamalage A. C. G. Weerasekara S. Hua D. H. Groutas W. C.. (2016). Reversal of the Progression of Fatal Coronavirus Infection in Cats by a Broad-Spectrum Coronavirus Protease Inhibitor. PloS Pathog. 12, 1–18. 10.1371/journal.ppat.1005531 PMC4814111 27027316

[B52] Kirchdoerfer R. N. Cottrell C. A. Wang N. S. Pallesen J. Yassine H. M. Turner H. L.. (2016). Pre-Fusion Structure of a Human Coronavirus Spike Protein. Nature 531, 118–121. 10.1038/nature17200 26935699 PMC4860016

[B53] Kirchdoerfer R. N. Ward A. B. (2019). Structure of the SARS-CoV nsp12 Polymerase Bound to Nsp7 and Nsp8 Co-Factors. Nat. Commun. 10, 1–9. 10.1038/s41467-019-10280-3 31138817 PMC6538669

[B54] Korber B. Fischer W. M. Gnanakaran S. Yoon H. Theiler J. Abfalterer W.. (2020). Tracking Changes in SARS-CoV-2 Spike: Evidence That D614G Increases Infectivity of the COVID-19 Virus. Cell 182, 812–827. 10.1016/j.cell.2020.06.043 32697968 PMC7332439

[B55] Kuba K. Imai Y. Penninger J. M. (2006). Angiotensin-Converting Enzyme 2 in Lung Diseases. Curr. Opin. Pharmacol. 6, 271–276. 10.1016/j.coph.2006.03.001 16581295 PMC7106490

[B56] Lan J. Ge J. Yu J. Shan S. Zhou H. Fan S.. (2020). Structure of the SARS-CoV-2 Spike Receptor-Binding Domain Bound to the ACE2 Receptor. Nature 581, 215–220. 10.1038/s41586-020-2180-5 32225176

[B57] Lehmann K. C. Gulyaeva A. Zevenhoven-Dobbe J. C. Janssen G. M. C. Ruben M. Overkleeft H. S.. (2015). Discovery of an Essential Nucleotidylating Activity Associated With a Newly Delineated Conserved Domain in the RNA Polymerase-Containing Protein of All Nidoviruses. Nucleic Acids Res. 43, 8416–8434. 10.1093/nar/gkv838 26304538 PMC4787807

[B58] Liang W. H. Lin Y. P. Bi J. P. Li J. F. Liang Y. Wong S. S.. (2020). Serosurvey of SARS-CoV-2 Among Hospital Visitors in China. Cell Res. 30, 817–818. 10.1038/s41422-020-0371-0 32686766 PMC7369564

[B59] Li W. H. Moore M. J. Vasilieva N. Sui J. H. Wong S. K. Berne M. A.. (2003). Angiotensin-Converting Enzyme 2 is a Functional Receptor for the SARS Coronavirus. Nature 426, 450–454. 10.1038/nature02145 14647384 PMC7095016

[B60] Ling R. Dai Y. Huang B. Huang W. Yu J. Lu X.. (2020). In Silico Design of Antiviral Peptides Targeting the Spike Protein of SARS-Cov-2. Peptides 130, 1–7. 10.1016/j.peptides.2020.170328 PMC7198429 32380200

[B61] Long Q. X. Liu B. Z. Deng H. J. Wu G. C. Deng K. Chen Y. K.. (2020). Antibody Responses to SARS-CoV-2 in Patients With COVID-19. Nat. Med. 26, 845–848. 10.1038/s41591-020-0897-1 32350462

[B62] Lu R. Zhao X. Li J. Niu P. Yang B. Wu H.. (2020). Genomic Characterisation and Epidemiology of 2019 Novel Coronavirus: Implications for Virus Origins and Receptor Binding. Lancet 395, 565–574. 10.1016/S0140-6736(20)30251-8 32007145 PMC7159086

[B63] Lv Z. Deng Y. Q. Ye Q. Cao L. Sun C. Y. Fan C.. (2020). Structural Basis for Neutralization of SARS-CoV-2 and SARS-CoV by a Potent Therapeutic Antibody. Science 369, 1505–1509. 10.1126/science.abc5881 32703908 PMC7402622

[B64] Lyon G. M. Mehta A. K. Varkey J. B. Brantly K. Plyler L. McElroy A. K.. (2014). Clinical Care of Two Patients With Ebola Virus Disease in the United States. N Engl. J. Med. 371, 2402–2409. 10.1056/NEJMoa1409838 25390460

[B65] Ma C. L. Sacco M. D. Hurst B. Townsend J. A. Hu Y. M. Szeto T.. (2020). Boceprevir, GC-376, and Calpain Inhibitors II, XII Inhibit SARS-CoV-2 Viral Replication by Targeting the Viral Main Protease. Cell Res. 30, 678–692. 10.1038/s41422-020-0356-z 32541865 PMC7294525

[B66] Millet J. K. Whittaker G. R. (2014). Host Cell Entry of Middle East Respiratory Syndrome Coronavirus After Two-Step, Furin-Mediated Activation of the Spike Protein. P Natl. Acad. Sci. U.S.A. 111, 15214–15219. 10.1073/pnas.1407087111 PMC4210292 25288733

[B67] Millet J. K. Whittaker G. R. (2015). Host Cell Proteases: Critical Determinants of Coronavirus Tropism and Pathogenesis. Virus Res. 202, 120–134. 10.1016/j.virusres.2014.11.021 25445340 PMC4465284

[B68] Mittal A. Manjunath K. Ranjan R. K. Kaushik S. Kumar S. Verma V. (2020). Covid-19 Pandemic: Insights Into Structure, Function, and Hace2 Receptor Recognition by the SARS-Cov-2. PloS Pathog. 16, 1–19. 10.20944/preprints202005.0260.v1 PMC7444525 32822426

[B69] Mulangu S. Dodd L. E. Davey R. T. Jr. Tshiani Mbaya O. Proschan M. Mukadi D.. (2019). A Randomized, Controlled Trial of Ebola Virus Disease Therapeutics. N Engl. J. Med. 281, 2293–2303. 10.1056/NEJMoa1910993 PMC10680050 31774950

[B70] Park J. E. Li K. Barlan A. Fehr A. R. Perlman S. McCray P. B.. (2016). Proteolytic Processing of Middle East Respiratory Syndrome Coronavirus Spikes Expands Virus Tropism. P Natl. Acad. Sci. U.S.A. 113, 12262–12267. 10.1073/pnas.1608147113 PMC5086990 27791014

[B71] Park K. S. Sun X. Aikins M. E. Moon J. J. (2020). Non-Viral COVID-19 Vaccine Delivery Systems. Adv. Drug Delivery Rev. 169, 137–151. 10.1016/j.addr.2020.12.008 PMC7744276 33340620

[B72] Pedersen N. C. Kim Y. Liu H. W. Kankanamalage A. C. G. Eckstrand C. Groutas W. C.. (2018). Efficacy of a 3C-Like Protease Inhibitor in Treating Various Forms of Acquired Feline Infectious Peritonitis. J. Feline Med. Surg. 20, 378–392. 10.1177/1098612x17729626 28901812 PMC5871025

[B73] Pinto D. Park Y. J. Beltramello M. Walls A. C. Tortorici M. A. Bianchi S.. (2020). Structural and Functional Analysis of a Potent Sarbecovirus Neutralizing Antibody. bioRxiv 2020.04.07.023903, 1–20. 10.1101/2020.04.07.023903

[B74] Plante J. A. Liu Y. Liu J. Xia H. Johnson B. A. Lokugamage K. G.. (2021). Spike Mutation D614G Alters SARS-CoV-2 Fitness. Nature 592, 116–121. 10.1038/s41586-020-2895-3 33106671 PMC8158177

[B75] Rabaan A. A. Al-Ahmed S. H. Haque S. Sah R. Tiwari R. Malik Y. S.. (2020). SARS-Cov-2, SARS-CoV, and MERS-COV: A Comparative Overview. Infez. Med. 28, 174–184. 32275259

[B76] Rainsford K. D. Parke A. L. Clifford-Rashotte M. Kean W. F. (2015). Therapy and Pharmacological Properties of Hydroxychloroquine and Chloroquine in Treatment of Systemic Lupus Erythematosus, Rheumatoid Arthritis and Related Diseases. Inflammopharmacology 5, 231–269. 10.1007/s10787-015-0239-y 26246395

[B77] Shang J. Ye G. Shi K. Wan Y. Luo C. Aihara H.. (2020). Structural Basis of Receptor Recognition by SARS-Cov-2. Nature 581, 221–224. 10.1038/s41586-020-2179-y 32225175 PMC7328981

[B78] Sharif-Askari N. S. Sharif-Askari F. S. Alabed M. Abou Tayoun A. Loney T. Uddin M.. (2020). Effect of Common Medications on the Expression of SARS-CoV-2 Entry Receptors in Kidney Tissue. Cts-Clin. Transl. Sci. 13, 1048–1054. 10.1111/cts.12862 PMC7461457 32799423

[B79] Sheahan T. P. Sims A. C. Graham R. L. Menachery V. D. Gralinski L. E. Case J. B.. (2017). Broad-Spectrum Antiviral GS-5734 Inhibits Both Epidemic and Zoonotic Coronaviruses. Sci. Transl. Med. 9, 1–12. 10.1126/scitranslmed.aal3653 PMC5567817 28659436

[B80] Sheahan T. P. Sims A. C. Zhou S. Graham R. L. Pruijssers A. J. Agostini M. L.. (2020). An Orally Bioavailable Broad-Spectrum Antiviral Inhibits SARS-CoV-2 in Human Airway Epithelial Cell Cultures and Multiple Coronaviruses in Mice. Sci. Transl. Med. 12, 1–15. 10.1126/scitranslmed.abb5883 PMC7164393 32253226

[B81] Shi R. Shan C. Duan X. Chen Z. Liu P. Song J.. (2020). A Human Neutralizing Antibody Targets the Receptor-Binding Site of SARS-Cov-2. Nature 584, 120–124. 10.1038/s41586-020-2381-y 32454512

[B82] Silveira M. M. Moreira G. Mendonca M. (2020). DNA Vaccines Against COVID-19: Perspectives and Challenges. Life Sci. 267, 1–7. 10.1016/j.lfs.2020.118919 PMC7749647 33352173

[B83] Song Z. Q. Xu Y. F. Bao L. L. Zhang L. Yu P. Qu Y. J.. (2019). From SARS to MERS, Thrusting Coronaviruses Into the Spotlight. Viruses-Basel 11, 1–28. 10.3390/v11010059 PMC6357155 30646565

[B84] South A. M. Tomlinson L. Edmonston D. Hiremath S. Sparks M. A. (2020). Controversies of Renin-Angiotensin System Inhibition During the COVID-19 Pandemic. Nat. Rev. Nephrol. 16, 305–307. 10.1038/s41581-020-0279-4 32246101 PMC7118703

[B85] Stas P. Faes D. Noyens P. (2008). Conduction Disorder and QT Prolongation Secondary to Long-Term Treatment With Chloroquine. Int. J. Cardiol. 2, e80–e82. 10.1016/j.ijcard.2007.04.055 17590456

[B86] Subissi L. Posthuma C. C. Collet A. Zevenhoven-Dobbe J. C. Gorbalenya A. E. Decroly E.. (2014). One Severe Acute Respiratory Syndrome Coronavirus Protein Complex Integrates Processive RNA Polymerase and Exonuclease Activities. P Natl. Acad. Sci. U.S.A. 111, E3900–E3909. 10.1073/pnas.1323705111 PMC4169972 25197083

[B87] Su S. Wong G. Shi W. Liu J. Lai A. C. K. Zhou J.. (2016). Epidemiology, Genetic Recombination, and Pathogenesis of Coronaviruses. Trends Microbiol. 24, 490–502. 10.1016/j.tim.2016.03.003 27012512 PMC7125511

[B88] Tchesnokov E. P. Feng J. Y. Porter D. P. Gotte M. (2019). Mechanism of Inhibition of Ebola Virus RNA-Dependent Rna Polymerase by Remdesivir. Viruses 11, 1–16. 10.3390/v11040326 PMC6520719 30987343

[B89] te Velthuis A. J. W. (2014). Common and Unique Features of Viral RNA-dependent Polymerases. Cell Mol. Life Sci. 71, 4403–4420. 10.1007/s00018-014-1695-z 25080879 PMC4207942

[B90] Tian X. Li C. Huang A. Xia S. Lu S. Shi Z.. (2020). Potent Binding of 2019 Novel Coronavirus Spike Protein by a SARS Coronavirus-Specific Human Monoclonal Antibody. Emerg. Microbes Infect. 9, 382–385. 10.1080/22221751.2020.1729069 32065055 PMC7048180

[B91] Toelzer C. Gupta K. Yadav S. K. N. Borucu U. Davidson A. D. Kavanagh Williamson M.. (2020). Free Fatty Acid Binding Pocket in the Locked Structure of SARS-CoV-2 Spike Protein. Science 370, 725–730. 10.1126/science.abd3255 32958580 PMC8050947

[B92] Vaduganathan M. Vardeny O. Michel T. McMurray J. J. V. Pfeffer M. A. Solomon S. D. (2020). Renin-Angiotensin-Aldosterone System Inhibitors in Patients With Covid-19. N Engl. J. Med. 382, 1653–1659. 10.1056/NEJMsr2005760 32227760 PMC7121452

[B93] Vaheri A. Strandin T. Hepojoki J. Sironen T. Henttonen H. Makela S.. (2013). Uncovering the Mysteries of Hantavirus Infections. Nat. Rev. Microbiol. 11, 539–550. 10.1038/nrmicro3066 24020072

[B94] van Hemert M. J. van den Worm S. H. Knoops K. Mommaas A. M. Gorbalenya A. E. Snijder E. J. (2008). SARS-Coronavirus Replication/Transcription Complexes are Membrane-Protected and Need a Host Factor for Activity In Vitro. PloS Pathog. 4, 1–10. 10.1371/journal.ppat.1000054 PMC2322833 18451981

[B95] Velthuis A. J. W. T. Arnold J. J. Cameron C. E. van den Worm S. H. E. Snijder E. J. (2011). The RNA Polymerase Activity of SARS-coronavirus nsp12 is Primer Dependent (Vol 38, Pg 203, 2010). Nucleic Acids Res. 39, 203–214. 10.1093/nar/gkr963 PMC2800238 19875418

[B96] Venkataraman S. Prasad B. V. L. S. Selvarajan R. (2018). Rna Dependent Rna Polymerases: Insights From Structure, Function and Evolution. Viruses-Basel 10, 1–23. 10.3390/v10020076 PMC5850383 29439438

[B97] Vincent M. J. Bergeron E. Benjannet S. Erickson B. R. Rollin P. E. Ksiazek T. G.. (2005). Chloroquine is a Potent Inhibitor of SARS Coronavirus Infection and Spread. Virol. J. 69, 1–10. 10.1186/1743-422X-2-69 PMC1232869 16115318

[B98] Walls A. C. Park Y. J. Tortorici M. A. Wall A. McGuire A. T. Veesler D. (2020). Structure, Function, and Antigenicity of the SARS-CoV-2 Spike Glycoprotein. Cell 181, 281–292. 10.1016/j.cell.2020.02.058 32155444 PMC7102599

[B99] Walls A. C. Tortorici M. A. Bosch B. J. Frenz B. Rottier P. J. M. DiMaio F.. (2016). Cryo-Electron Microscopy Structure of a Coronavirus Spike Glycoprotein Trimer. Nature 531, 114–117. 10.1038/nature16988 26855426 PMC5018210

[B100] Wang M. L. Cao R. Y. Zhang L. K. Yang X. L. Liu J. Xu M. Y.. (2020). Remdesivir and Chloroquine Effectively Inhibit the Recently Emerged Novel Coronavirus, (2019-nCoV) In Vitro. Cell Res. 30, 269–271. 10.1038/s41422-020-0282-0 32020029 PMC7054408

[B101] Wang F. Chen C. Tan W. Yang K. Yang H. (2016). Structure of Main Protease From Human Coronavirus NL63: Insights for Wide Spectrum Anti-Coronavirus Drug Design. Sci. Rep. 6, 1–12. 10.1038/srep22677 26948040 PMC4780191

[B102] Wang F. H. Chen C. Yang K. L. Xu Y. Liu X. M. Gao F.. (2017). Michael Acceptor-Based Peptidomimetic Inhibitor of Main Protease From Porcine Epidemic Diarrhea Virus. J. Med. Chem. 60, 3212–3216. 10.1021/acs.jmedchem.7b00103 28287727

[B103] Wang K. Chen W. Zhang Z. Deng Y. Lian J. Q. Du P.. (2020). CD147-Spike Protein is a Novel Route for SARS-CoV-2 Infection to Host Cells. Signal Transduct. Target Ther. 5, 1–10. 10.1038/s41392-020-00426-x 33277466 PMC7714896

[B104] Wong R. S. Y. (2020). The SARS-CoV-2 Outbreak: An Epidemiological and Clinical Perspective. SN Compr. Clin. Med. 29, 1–9. 10.1007/s42399-020-00546-z PMC7524027 33015553

[B105] Woo P. C. Y. Lau S. K. P. Lam C. S. F. Lau C. C. Y. Tsang A. K. L. Lau J. H. N.. (2012). Discovery of Seven Novel Mammalian and Avian Coronaviruses in the Genus Deltacoronavirus Supports Bat Coronaviruses as the Gene Source of Alphacoronavirus and Betacoronavirus and Avian Coronaviruses as the Gene Source of Gammacoronavirus and Deltacoronavirus. J. Virol. 86, 3995–4008. 10.1128/Jvi.06540-11 22278237 PMC3302495

[B106] Wrapp D. Wang N. Corbett K. S. Goldsmith J. A. Hsieh C. L. Abiona O.. (2020). Cryo-Em Structure of the 2019-Ncov Spike in the Prefusion Conformation. Science 367, 1260–1263. 10.1126/science.abb2507 32075877 PMC7164637

[B107] Wu K. L. Chen L. Peng G. Q. Zhou W. B. Pennell C. A. Mansky L. M.. (2011). A Virus-Binding Hot Spot on Human Angiotensin-Converting Enzyme 2 Is Critical for Binding of Two Different Coronaviruses. J. Virol. 85, 5331–5337. 10.1128/Jvi.02274-10 21411533 PMC3094985

[B108] Wu Z. Y. McGoogan J. M. (2020). Characteristics of and Important Lessons From the Coronavirus Disease 2019 (Covid-19) Outbreak in China Summary of a Report of 72 314 Cases From the Chinese Center for Disease Control and Prevention. Jama-J. Am. Med. Assoc. 323, 1239–1242. 10.1001/jama.2020.264810.1001/jama.2020.2648 32091533

[B109] Wu N. C. Yuan M. Liu H. Lee C. D. Zhu X. Bangaru S.. (2020). An Alternative Binding Mode of IGHV3-53 Antibodies to the SARS-CoV-2 Receptor Binding Domain. Cell Rep. 33, 1–8. 10.1016/j.celrep.2020.108274 PMC7522650 33027617

[B110] Wu F. Zhao S. Yu B. Chen Y.-M. Wang W. Hu Y.. (2020a). Complete Genome Characterisation of a Novel Coronavirus Associated With Severe Human Respiratory Disease in Wuhan, China. bioRxiv 2020, 1–33. 10.1101/2020.01.24.919183

[B111] Wu F. Zhao S. Yu B. Chen Y. M. Wang W. Song Z. G.. (2020b). A New Coronavirus Associated With Human Respiratory Disease in China. Nature 579, 265–269. 10.1038/s41586-020-2202-3 32015508 PMC7094943

[B112] Xia S. Liu Q. Wang Q. Sun Z. W. Su S. Dub L. Y.. (2014). Middle East Respiratory Syndrome Coronavirus (MERS-CoV) Entry Inhibitors Targeting Spike Protein. Virus Res. 194, 200–210. 10.1016/j.virusres.2014.10.007 25451066 PMC7114414

[B113] Xia S. Liu M. Q. Wang C. Xu W. Lan Q. S. Feng S. L.. (2020). Inhibition of SARS-CoV-2 (Previously 2019-nCoV) Infection by a Highly Potent Pan-Coronavirus Fusion Inhibitor Targeting its Spike Protein That Harbors a High Capacity to Mediate Membrane Fusion. Cell Res. 30, 343–355. 10.1038/s41422-020-0305-x 32231345 PMC7104723

[B114] Xiu S. Dick A. Ju H. Mirzaie S. Abdi F. Cocklin S.. (2020). Inhibitors of SARS-CoV-2 Entry: Current and Future Opportunities. J. Med. Chem. 63, 12256–12274. 10.1021/acs.jmedchem.0c00502 32539378 PMC7315836

[B115] Xue X. Y. Yu H. W. Yang H. T. Xue F. Wu Z. X. Shen W.. (2008). Structures of Two Coronavirus Main Proteases: Implications for Substrate Binding and Antiviral Drug Design. J. Virol. 82, 2515–2527. 10.1128/Jvi.02114-07 18094151 PMC2258912

[B116] Xu C. Wang Y. Liu C. Zhang C. Han W. Hong X.. (2020). Conformational Dynamics of SARS-CoV-2 Trimeric Spike Glycoprotein in Complex With Receptor ACE2 Revealed by Cryo-EM. Sci. Adv. 7, 1–13. 10.1126/sciadv.abe5575 PMC7775788 33277323

[B117] Xu J. Zhao S. Teng T. Abdalla A. E. Zhu W. Xie L.. (2020). Systematic Comparison of Two Animal-to-Human Transmitted Human Coronaviruses: Sars-CoV-2 and SARS-Cov. Viruses 12, 1–17. 10.3390/v12020244 PMC7077191 32098422

[B118] Yang H. Xie W. Xue X. Yang K. Ma J. Liang W.. (2005). Design of Wide-Spectrum Inhibitors Targeting Coronavirus Main Proteases. PloS Biol. 3, 1742–1752. 10.1371/journal.pbio.0030324 PMC1197287 16128623

[B119] Yan R. Zhang Y. Li Y. Xia L. Guo Y. Zhou Q. (2020). Structural Basis for the Recognition of SARS-CoV-2 by Full-Length Human ACE2. Science 367, 1444–1448. 10.1126/science.abb2762 32132184 PMC7164635

[B120] Yao H. Lu X. Chen Q. Xu K. Chen Y. Cheng L.. (2020a). Patient-Derived Mutations Impact Pathogenicity of SARS-Cov-2. medRxiv 2020, 1–57. 10.1101/2020.04.14.20060160

[B121] Yao H. Song Y. Chen Y. Wu N. Xu J. Sun C.. (2020b). Molecular Architecture of the SARS-CoV-2 Virus. Cell 183, 730–738. 10.1016/j.cell.2020.09.018 32979942 PMC7474903

[B122] Yao X. Ye F. Zhang M. Cui C. Huang B. Niu P.. (2020). In Vitro Antiviral Activity and Projection of Optimized Dosing Design of Hydroxychloroquine for the Treatment of Severe Acute Respiratory Syndrome Coronavirus 2 (SARS-Cov-2). Clin. Infect. Dis. 15, 732–739. 10.1093/cid/ciaa237 PMC7108130 32150618

[B123] Yaylali S. A. Sadigov F. Erbil H. Ekinci A. Akcakaya A. A. (2013). Chloroquine and Hydroxychloroquine Retinopathy-Related Risk Factors in a Turkish Cohort. Int. Ophthalmol. 33, 627–634. 10.1007/s10792-013-9748-0 23456514

[B124] Yin W. Mao C. Luan X. Shen D. D. Shen Q. Su H.. (2020). Structural Basis for Inhibition of the RNA-dependent RNA Polymerase From SARS-CoV-2 by Remdesivir. Science 368, 1499–1504. 10.1126/science.abc1560 32358203 PMC7199908

[B125] Yuan M. Liu H. Wu N. C. Lee C. D. Zhu X. Zhao F.. (2020a). Structural Basis of a Shared Antibody Response to SARS-Cov-2. Science 369, 1119–1123. 10.1126/science.abd2321 32661058 PMC7402627

[B126] Yuan M. Wu N. C. Zhu X. Lee C. D. So R. T. Y. Lv H.. (2020b). A Highly Conserved Cryptic Epitope in the Receptor Binding Domains of SARS-CoV-2 and SARS-Cov. Science 368, 630–633. 10.1126/science.abb7269 32245784 PMC7164391

[B127] Yurkovetskiy L. Wang X. Pascal K. E. Tomkins-Tinch C. Nyalile T. P. Wang Y.. (2020). Structural and Functional Analysis of the D614G SARS-Cov-2 Spike Protein Variant. Cell 183, 739–751. 10.1016/j.cell.2020.09.032 32991842 PMC7492024

[B128] Zaki A. M. van Boheemen S. Bestebroer T. M. Osterhaus A. D. Fouchier R. A. (2012). Isolation of a Novel Coronavirus From a Man With Pneumonia in Saudi Arabia. N Engl. J. Med. 367, 1814–1820. 10.1056/NEJMoa1211721 23075143

[B129] Zhang L. L. Lin D. Z. Kusov Y. Nian Y. Ma Q. J. Wang J.. (2020). alpha-Ketoamides as Broad-Spectrum Inhibitors of Coronavirus and Enterovirus Replication: Structure-Based Design, Synthesis, and Activity Assessment. J. Med. Chem. 63, 4562–4578. 10.1021/acs.jmedchem.9b01828 32045235

[B130] Zhou D. Duyvesteyn H. M. E. Chen C. P. Huang C. G. Chen T. H. Shih S. R.. (2020). Structural Basis for the Neutralization of SARS-CoV-2 by an Antibody From a Convalescent Patient. Nat. Struct. Mol. Biol. 27, 950–958. 10.1038/s41594-020-0480-y 32737466

[B131] Zhou P. Fan H. Lan T. Yang X. L. Shi W. F. Zhang W.. (2018). Fatal Swine Acute Diarrhoea Syndrome Caused by an HKU2-related Coronavirus of Bat Origin. Nature 556, 255–258. 10.1038/s41586-018-0010-9 29618817 PMC7094983

[B132] Zhu Y. Yu D. Yan H. Chong H. He Y. (2020). Design of Potent Membrane Fusion Inhibitors Against SARS-CoV-2, an Emerging Coronavirus With High Fusogenic Activity. J. Virol. 94, 1–12. 10.1128/JVI.00635-20 PMC7343218 32376627

[B133] Ziebuhr J. (2005). The Coronavirus Replicase. Curr. Top. Microbiol. Immunol. 287, 57–94. 10.1007/3-540-26765-4_3 15609509 PMC7121973

[B134] Ziebuhr J. Snijder E. J. Gorbalenya A. E. (2000). Virus-Encoded Proteinases and Proteolytic Processing in the Nidovirales. J. Gen. Virol. 81, 853–879. 10.1099/0022-1317-81-4-853 10725411

[B135] Zumla A. Hui D. S. Azhar E. I. Memish Z. A. Maeurer M. (2020). Reducing Mortality From 2019-nCoV: Host-Directed Therapies Should be an Option. Lancet 395, e35–e36. 10.1016/S0140-6736(20)30305-6 32035018 PMC7133595

