# Supplementary material for: SARS-CoV-2: Origin, Evolution, and Targeting Inhibition
Source: Front Cell Infect Microbiol. 2021 Jun 17;11:676451. doi: 10.3389/fcimb.2021.676451 (PMC8248807; doi:10.3389/fcimb.2021.676451)
Supplement: Supplementary file 1 [file DataSheet_1.pdf]

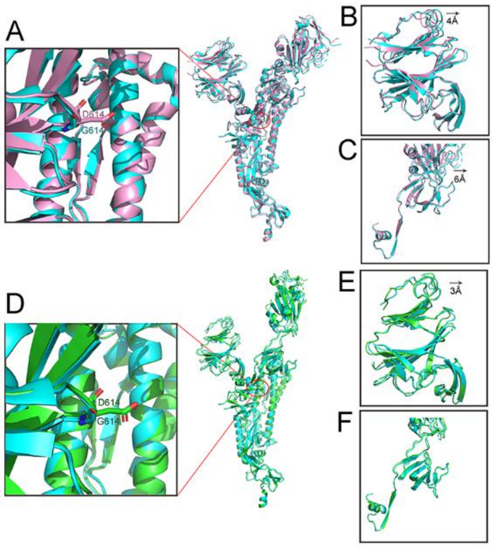

Supplementary Figure 1. Structural differences between ancestral spike and mutant D614G

(A) The overall structural differences between ancestral spike (pink) and mutant D614G (cyan) in the closed state. **(B)** Comparison of the NTD of S1 subunit between ancestral spike (pink) and mutant D614G (cyan) in the closed state. **(C)** Comparison of the INT of S1 subunit between ancestral spike (pink) and mutant D614G (cyan) in the closed state. (D) The overall structural differences between ancestral spike (green) and mutant D614G (cyan)in the open state. (E) Comparison of the NTD of S1 subunit between ancestral spike (green) and mutant D614G (cyan) in the open state. (F) Comparison of the INT of S1 subunit between ancestral spike (green) and mutant D614G (cyan) in the open state. Arrows indicate the relative movement of the S1 subunit between ancestral spike and mutant D614G. All structures are drawn by Pymol.

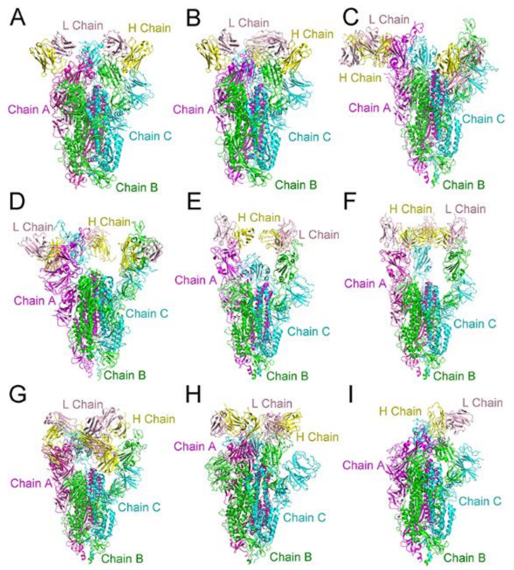

Supplementary Figure 2. Complex structure of S protein with antibodies.
(A) Complex structure of S protein with two s309 Fabs. S protein, chain A and chain B, bind with two s309 Fabs. RBD in chain C is shown in open state without binding with s309 Fab. **(B)** Complex structure of S protein with three s309 Fabs. All RBD of S protein is shown in closed state. **(C)** Complex structure of S protein with two H014 Fabs. S protein, chain A and chain B, bind with two H014 Fabs. RBD in chain C is shown in closed state without binding with H014 Fab. **(D)** Complex structure of S protein with three H014 Fab. All RBD of S protein is shown in open state. **(E)** Complex structure of S protein with two C105 Fabs. S protein, chain A and chain B, bind with two C105 Fabs. RBD in chain C is shown in closed state without binding with C105 Fab. **(F)** Complex structure of S protein with three C105 Fabs. All RBD of S protein is shown in open state. **(G)** Complex structure of S protein with two EY6A Fab. **(H)** Complex structure of S protein with two Fab2-4. **(I)** Complex structure of S protein with BD-23 Fab. The three protomers in the S trimer are shown in cyan, green, and magnet, respectively. Neutralizing antibodies is colored in yellow (heavy chain) and pink (light chain) in each figure. All structures are drawn by Pymol.

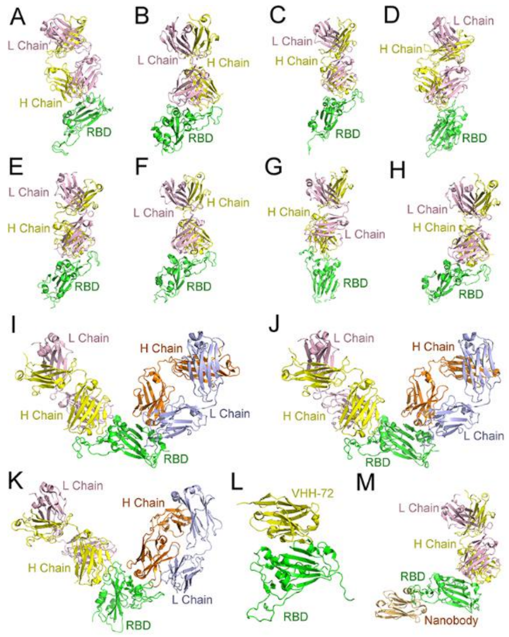

Supplementary Figure 3. Complex structure of RBD with antibodies.

(A) Complex structure of RBD with CR3022 Fab. **(B)** Complex structure of RBD with CC12.1. **(C)** Complex structure of RBD with CC12.3. **(D)** Complex structure of RBD with P2B-2F6. **(E)** Complex structure of RBD with CB6. **(F)** Complex structure of RBD with CV30 Fab. **(G)** Complex structure of RBD with COVA2-39. **(H)** Complex structure of RBD with COVA2-04. Above all, neutralizing antibodies is colored in yellow (heavy chain) and pink (light chain) in each figure. **(I)** Complex structure of RBD with CC12.1 and CR3022. Neutralizing antibodies, CC12.1, is colored in yellow (heavy chain) and pink (light chain). CR3022 is colored in orange (heavy chain) and light purple (light chain). **(J)** Complex structure of RBD with CC12.3 and CR3022. Neutralizing antibodies, CC12.3, is colored in yellow (heavy chain) and pink (light chain). CR3022 is colored in orange (heavy chain) and light purple (light chain). **(K)** Complex structure of RBD with REGN 10933 and REGN 10987. Neutralizing antibodies, REGN 10987, is colored in yellow (heavy chain) and pink (light chain). REGN 10933 is colored in orange (heavy chain) and light purple (light chain). **(L)** Complex structure of RBD with VHH-72. Neutralizing antibody, VHH-72, is colored in yellow. **(M)** Complex structure of RBD with EY6A Fab and nanobody. Neutralizing antibodies, EY6A Fab, is colored in yellow (heavy chain) and pink (light chain). Nanobody is colored in orange. The RBD is shown in green in all figures. All structures are drawn by Pymol.
